# Oxidation-activated nanotherapy boosts tumor immunity and disrupts tumor-nerve crosstalk to combat bone metastases and cancer pain

**DOI:** 10.1126/sciadv.ady1292

**Published:** 2026-01-23

**Authors:** Zhaowei Zhang, Pengfei Chen, Yufei Zheng, Mobai Li, Lan Zhao, Zezhou Fu, Yujie Zhou, Tingyu Zhang, Xuanrong Sun, Dingcheng Zhu, Youqing Shen, Shunwu Fan, Xin Liu, Jiajia Xiang

**Affiliations:** ^1^ Department of Orthopaedic Surgery, Sir Run Run Shaw Hospital, Zhejiang University School of Medicine, Hangzhou 310016, China.; ^2^ Key Laboratory of Mechanism Research and Precision Repair of Orthopaedic Trauma and Aging Diseases of Zhejiang Province, Hangzhou 310016, China.; ^3^ Zhejiang Key Laboratory of Smart Biomaterials and Center for Bionanoengineering, College of Chemical and Biological Engineering, Zhejiang University, Hangzhou 310058, China.; ^4^ Key Laboratory of Biomass Chemical Engineering of the Ministry of Education, College of Chemical and Biological Engineering, Zhejiang University, Hangzhou 310058, China.; ^5^ Collaborative Innovation Center of Yangtze River Delta Region Green Pharmaceuticals, Zhejiang University of Technology, Hangzhou 310014, China.; ^6^ College of Material, Chemistry, and Chemical Engineering, Key Laboratory of Organosilicon Chemistry and Material Technology, Ministry of Education, Hangzhou Normal University, Hangzhou 311121, China.

## Abstract

Bone metastasis remains a formidable challenge in oncology due to the interdependent triad of immunosuppression, neuropathic pain, and osteolytic destruction. Current treatments fail to holistically address these pathophysiological axes. Here, we develop a reactive oxygen species (ROS)–responsive liposomal nanoplatform (LipoNCs@pGSDMB) that codelivers a polymeric stimulator of interferon genes (STING) agonist and a gasdermin B (GSDMB) plasmid for dual neuro-immune modulation. Upon tumor-selective activation in metastatic bone niches, this nanotherapy induces STING-driven immune priming and GSDMB-mediated pyroptosis, triggering potent antitumor responses. Crucially, LipoNCs@pGSDMB restore voltage-gated calcium channel (VGCC) expression in tumor cells, a prognostic biomarker identified through multiomics analysis of clinical specimens, thereby blocking calcium-dependent neurosignaling and disrupting prometastatic tumor-nerve cross-talk. In breast cancer bone metastasis models, this approach achieves 94% tumor suppression, complete pain resolution, and efficient bone restoration. By converging oxidation-responsive nanomaterial engineering, immunomodulation, and neural circuit reprogramming, this work establishes a paradigm-shifting neuroimmunotherapy platform that dismantles the self-reinforcing metastasis niche while addressing its debilitating sequelae.

## Teaser

A nanotherapy that reprograms immune and neural circuits halts bone metastasis, relieves cancer pain, and restores bone integrity.

## INTRODUCTION

Bone metastasis represents a lethal manifestation of advanced malignancies, affecting more than 70% of patients with metastatic breast or prostate cancer (*1*). Despite some progress in treatment, median survival remains limited to 2 to 3 years, with 5-year survival rates dropping below 10% (*2*, *3*). Current therapies modestly delay skeletal-related events (SREs), including osteolytic destruction and pathological fractures, but fail to address the complex and dynamic interactions within the bone microenvironment that drive metastatic progression (*4*). The bone is a vascularized and innervated niche that supports tumor colonization and sustains disease through intricate cross-talk with bone-resident cells (*5*). Effective treatment requires strategies that disrupt this adaptive ecosystem.

A defining feature of bone metastasis is its extensive neural involvement within the tumor microenvironment (TME). Bone metastases—especially those arising from breast, prostate, and lung cancers—exhibit increased innervation (*6*, *7*) and reciprocal communication with both sensory and sympathetic nerves (*8*). Tumor cells secrete neurotrophic factors such as nerve growth factor and brain-derived neurotrophic factor, which stimulate nerve sprouting, sensitization, and chronic pain (*9*, *10*). In return, tumor-educated nerves release neurotransmitters such as norepinephrine and substance P, promoting tumor proliferation, angiogenesis, and osteoclastogenesis (*11*, *12*). This ongoing signaling loop accelerates tumor progression and correlates with poor clinical outcomes. Current pain management strategies, including those outlined by the World Health Organization (WHO), often fail to effectively control neuropathic pain (*13*). Worse still, opioid analgesics carry risks of addiction and exacerbate immunosuppression (*14*). Moreover, unresolved tumor-nerve interactions, even after effective tumor control, fuel tumor recurrence and chronic pain (*15*, *16*). This reinforces the need for therapies that disrupt this tumor-nerve cross-talk, a challenge that has yet to be addressed by existing treatments (*17*–*20*).

In addition to neural involvement, bone metastases are characterized by a profoundly immunosuppressive tumor microenvironment (TIME) that allows tumor cells to evade immune surveillance (*10*, *21*). Metastatic cells often inherit immunoevasive traits from their primary tumors, including low mutation burdens and defective immune infiltration (*22*). The bone marrow niche further reinforces this suppression by recruiting myeloid-derived suppressor cells (MDSCs), regulatory T cells (T_reg_ cells), and M2-polarized tumor-associated macrophages (TAMs) (*23*, *24*). Moreover, tumor-nerve cross-talk compounds this immunosuppressive environment. Neurotransmitters such as norepinephrine and acetylcholine promote the accumulation of MDSCs and T_reg_ cells, polarize TAMs toward an immunosuppressive phenotype, and impair cytotoxic T lymphocytes (CTLs) and natural killer (NK) cells (*25*–*27*). Meanwhile, calcitonin gene–related peptide (CGRP) inhibits dendritic cell (DC) maturation and accelerates CTL exhaustion (*28*, *29*). In parallel, infiltrating immune cells secrete inflammatory mediators that further sensitize nerves, reinforcing tumor-nerve interactions and promoting immune evasion (*30*). This dynamic interaction between immune suppression and tumor-nerve signaling exacerbates tumor growth and metastasis. Recent preclinical studies have shown promise in reshaping the TIME through STING activation and pyroptosis induction (*31*, *32*). STING agonists stimulate type I interferon (IFN-I) responses, driving DC maturation and tumor antigen presentation, while pyroptosis releases tumor antigens and amplifies immune activation. However, these strategies fail to address the role of neural circuits in sustaining metastasis.

**Fig. 1.**
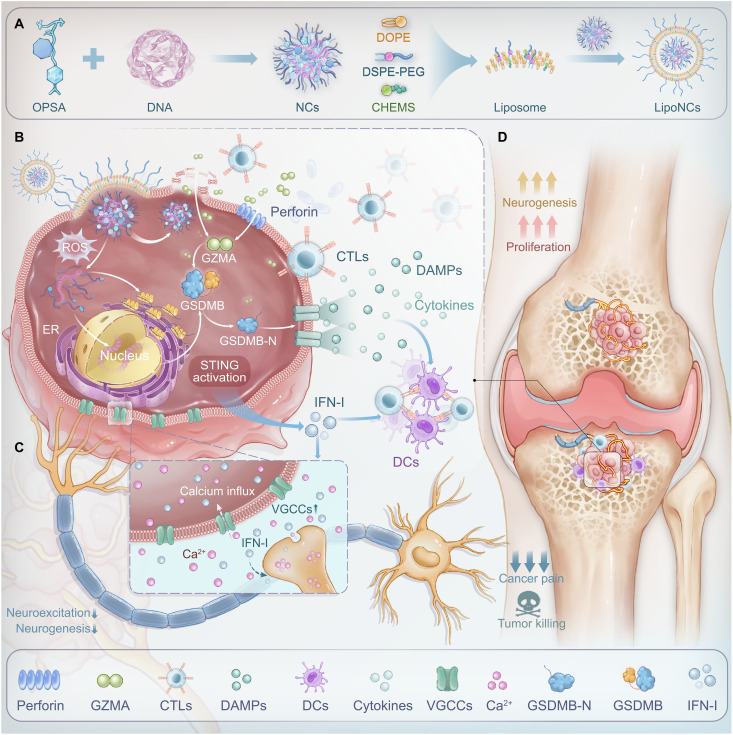
Schematic illustration of immune and nerve modulation mediated by LipoNCs@pGSDMB for enhanced bone metastasis therapy. (**A**) Oxidation-responsive derivative (OPSA) and plasmid DNA form NCs, which are subsequently encapsulated within liposomes to generate LipoNCs. (**B**) After internalization by tumor cells via membrane fusion, LipoNCs@pGSDMB activate the STING pathway and induce GSDMB-mediated pyroptosis through immune cell engagement, initiating robust antitumor immune responses. (**C**) LipoNCs@pGSDMB up-regulate VGCCs in tumor cells, thereby increasing calcium influx and disrupting tumor-nerve cross-talk. (**D**) LipoNCs@pGSDMB exhibit potent and holistic antitumor efficacy against bone metastases, addressing both tumor progression and its associated cancer pain. GAPDH, glyceraldehyde-3-phosphate dehydrogenase; DOPE, 1,2-dioleoyl-*sn*-glycero-3-phosphoethanolamine; CHEMS, cholesteryl hemisuccinate; DPSE-PEG, 1,2-distearoyl-*sn*-glycero-3-phosphoethanolamine *N*-[methoxy(polyethylene glycol)-2000]; DAMPS, damage-associated molecular patterns; CTLs, cytotoxic T lymphocytes; IFN-I, type I interferon; DCs, dendritic cells; GZMA, granzyme A.

Targeting the neuro-immune-metastasis axis presents a promising therapeutic opportunity for intervention. Monotherapies targeting a single pathway are unlikely to overcome the adaptive nature of this complex network. To address this challenge, we develop LipoNCs@pGSDMB, a tumor-selective nanoplatform that harnesses the elevated reactive oxygen species (ROS) levels in bone metastases to spatiotemporally release a polymeric STING agonist (PSA) and gasdermin B plasmid (pGSDMB) (Fig. 1). This nanotherapy activates the STING pathway and induces pyroptosis, eliciting robust antitumor immune responses. Crucially, LipoNCs@pGSDMB restore voltage-gated calcium channel (VGCC) expression in tumor cells (*33*), identified as a prognostic biomarker via multiomics analysis, disrupting calcium-dependent neurosignaling (*33*) and breaking tumor-nerve cross-talk. In murine models, this approach effectively eradicates tumors, reverses osteolysis, and alleviates cancer pain. By integrating immunomodulation, neural circuit reprogramming, and ROS-responsive nanomaterial engineering, our study establishes a neuroimmunotherapy paradigm capable of addressing the multifaceted challenges of bone metastasis treatment.

## RESULTS

### Dual-acting NCs for STING activation and pyroptosis induction

**Fig. 2.**
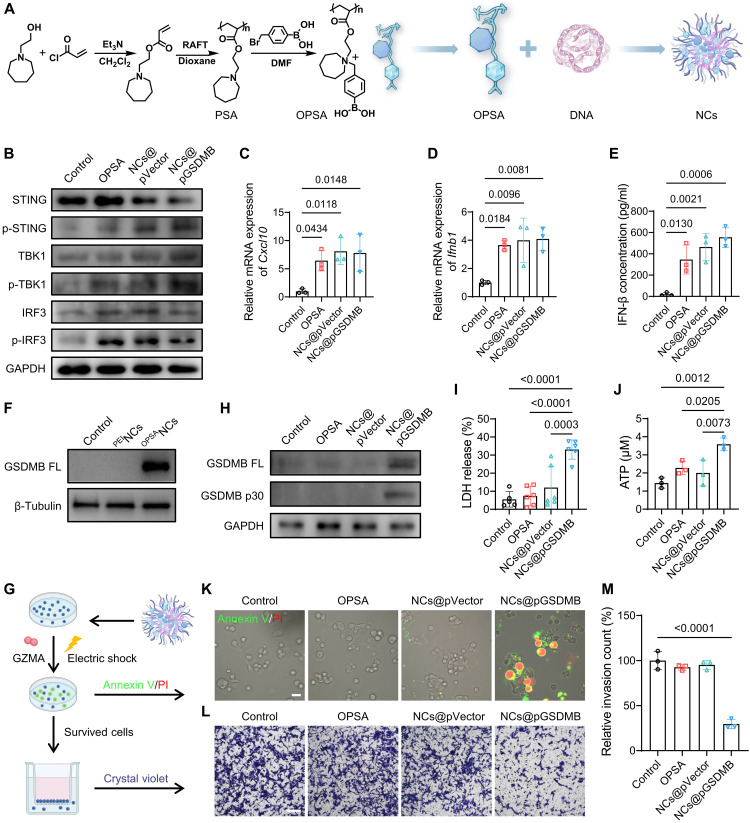
Preparation and characterization of the STING-activating and pyroptosis-inducing NCs. (**A**) Schematic illustration of OPSA synthesis and its self-assembly with DNA into NCs. (**B**) Expression and phosphorylation levels of STING, TBK1, and IRF3 in 4T1 cells across different treatments. (**C** and **D**) mRNA expression of *Cxcl10* (C) and *Ifnb1* (D) in 4T1 cells. (**E**) Quantification of IFN-β levels in the supernatant of 4T1 cells after treatments. (**F**) GSDMB protein overexpression in 4T1 cells treated with ^OPSA^NCs@pGSDMB or ^PEI^NCs@pGSDMB. (**G**) Schematic illustration of pyroptosis simulation via electroporation-mediated GZMA delivery and subsequent invasion assay in the Transwell device. Created in BioRender. Z. Zhang (2026); https://BioRender.com/u8kp77s. (**H**) Expression levels of full-length (FL) GSDMB and its cleaved p30 fragment in 4T1 cells postpyroptosis simulation. (**I** and **J**) Quantification of lactate dehydrogenase (LDH) (I) and adenosine triphosphate (ATP) (J) levels in the supernatant of 4T1 cells following the pyroptosis simulation. (**K**) Annexin V/propidium iodide (PI) staining images of 4T1 cells postpyroptosis simulation. Scale bar, 20 μm. (**L** and **M**) Crystal violet staining images (L) from invasion assays following pyroptosis simulation and corresponding quantification (M). Scale bar, 200 μm. Data are presented as means ± SD. Statistical comparisons among groups were performed using one-way analysis of variance (ANOVA). PSA, polymeric STING agonist.

To confront the challenges of activating antitumor immunity within the TIME, we engineered oxidation-responsive nanocomplexes (NCs) that activate the STING pathway and induce pyroptosis. The core component, poly(2-(azepan-1-yl)ethylacrylate), functions as a PSA with innate immunostimulatory potential (*34*). However, its hydrophobic nature and low cationic charge limited its DNA binding capability, constraining its gene delivery efficiency (fig. S1). To address these shortcomings, we chemically modified PSA via quaternization with 4-bromomethylphenylboronic acid, yielding an oxidation-responsive derivative (OPSA) with enhanced hydrophilicity and positive charge density (Fig. 2A and fig. S2). OPSA self-assembled with plasmid DNA into NCs with a diameter of around 40 nm and a ζ potential of +10 to 15 mV across a range of nitrogen to phosphorus (N/P) ratios (figs. S3 and S4), demonstrating robust DNA encapsulation.

The premise of this design lies in the charge removal and subsequent reversion of OPSA to its native PSA form upon exposure to ROS, a hallmark of the TME, where intracellular ROS concentrations in tumor cells can reach up to 100 μM (*35*). This redox-responsive behavior may facilitate DNA release and thus STING activation. Exposure to H_2_O_2_ induced conversion of quaternary ammonium groups in OPSA to tertiary amines, as indicated by the formation of *p*-hydroxymethylphenol, detected by high-performance liquid chromatography (HPLC) (fig. S5) and ^1^H nuclear magnetic resonance (fig. S6). This conversion shifted the ζ potential of OPSA NCs from +10 to −13 mV (fig. S7), indicating OPSA reversion to PSA. Transmission electron microscopy (TEM) and dynamic light scattering (DLS) revealed complete NC disintegration at 100 μM H_2_O_2_ (figs. S8 and S9), while gel electrophoresis confirmed ROS-dependent DNA release after 1-hour incubation with above 40 μM H_2_O_2_ (fig. S10). This ROS-triggered transition from hydrophilic OPSA to hydrophobic PSA potentially enables efficient intracellular gene delivery and STING activation.

The gene delivery performance of OPSA NCs was assessed in hard-to-transfect 4T1 cells using a luciferase reporter plasmid (pLuc). At an optimal N/P ratio of 15, OPSA NCs achieved transfection efficiencies four orders of magnitude higher than polyethyleneimine (PEI), a widely used gold standard polymeric vector (fig. S11), while maintaining more than 90% cell viability (fig. S12). This superiority was further amplified under TME-mimetic conditions at 100 μM H_2_O_2_ (fig. S13), where ROS-triggered NC disassembly enhanced DNA release. Notably, Cy5-labeled pLuc (^Cy5^pLuc)–loaded NCs demonstrated minimal lysosomal colocalization after a 4-hour incubation (fig. S14), indicating effective evasion of lysosomal degradation, a major bottleneck in nonviral gene delivery. Following ROS-responsive payload release, the liberated PSA polymer activated the STING pathway, as evidenced by phosphorylation of STING, interferon regulatory factor 3 (IRF3), and TANK-binding kinase 1 (TBK1) (Fig. 2B and fig. S15), the up-regulation of IFN-stimulated genes (*Cxcl10* and *Ifnb1*) (Fig. 2, C and D), and elevated IFN-β secretion (Fig. 2E). The STING activation effect was further validated in murine Lewis lung carcinoma (LLC) cell line, a representative model of lung cancer bone metastasis (fig. S16). STING activation was found to depend on ROS levels, reaching maximal induction at 100 μM H_2_O_2_ (fig. S17), and potential confounding effects of H_2_O_2_ were ruled out through appropriate control experiments (fig. S18). These findings underscore the tumor-selective immunostimulatory capabilities of OPSA NCs, exploiting pathological ROS levels to spatially restrict immune activation to malignant niches.

To integrate pyroptosis, we loaded OPSA NCs with a GSDMB plasmid (NCs@pGSDMB). GSDMB, a pore-forming protein activated by CTL-derived perforin and granzyme A (GZMA) (*36*), facilitates immunogenic cell death upon cleavage. NCs@pGSDMB markedly up-regulated GSDMB expression in 4T1 cells (Fig. 2F). To mimic immune cell-mediated activation, we either electroporated GZMA into 4T1 cells (*36*) or cocultured them with NK cells, which induced proteolytic cleavage of full-length GSDMB into its active N-terminal fragment (GSDMB-N) (Fig. 2, G and H, and fig. S19). This activation triggered hallmark pyroptotic events, including the rapid release of lactate dehydrogenase (LDH) and adenosine triphosphate (ATP) (Fig. 2, I and J), along with the formation of pyroptotic bodies visualized through annexin V/propidium iodide staining (Fig. 2K). This GSDMB-mediated pyroptosis was also observed in LLC cells (fig. S20). Moreover, the residual surviving cells exhibited significantly reduced invasiveness, as evidenced by Transwell assays (Fig. 2, L and M). These results demonstrate that OPSA NCs synergistically activate the STING pathway and GSDMB-induced immunogenic pyroptosis while simultaneously impairing tumor cell invasiveness, a critical step in limiting metastatic dissemination.

### Polymer-induced membrane perforation enhances cell pyroptosis

**Fig. 3.**
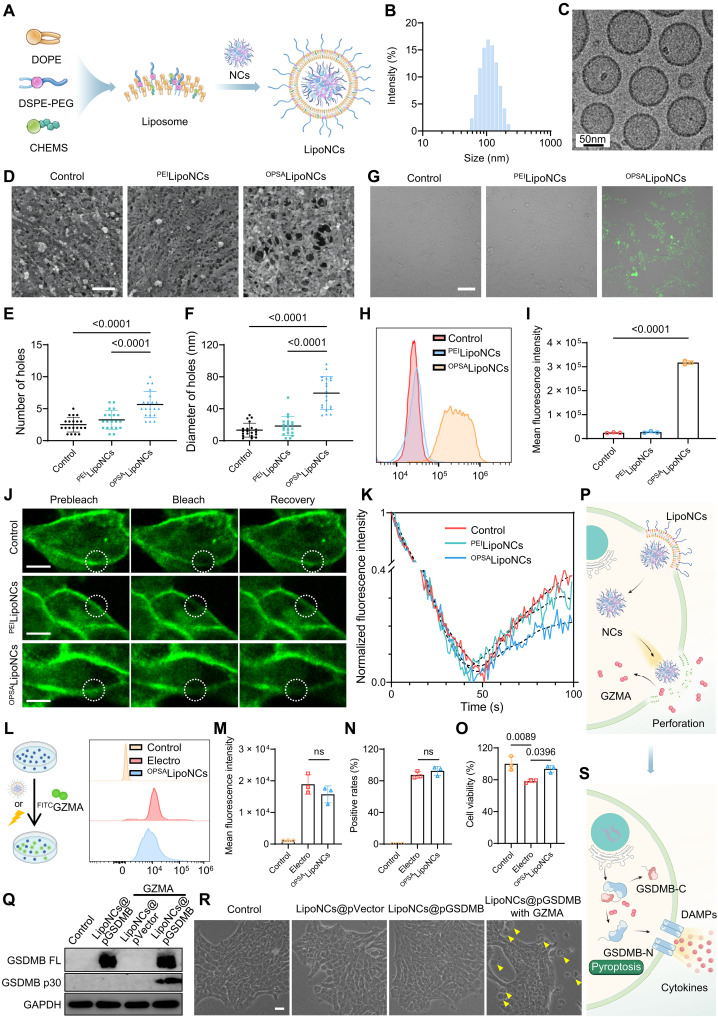
Preparation of LipoNCs and their pyroptosis-enhancing effects through membrane perforation. (**A**) Schematic representation of LipoNC preparation. NCs were enveloped with liposomes composed of DOPE, DSPE-PEG, and CHEMS. (**B**) Size distribution of LipoNCs as measured by DLS. (**C**) Representative cryo-TEM images showing the morphology of LipoNCs. Scale bar, 50 nm. (**D**) SEM images illustrating the surface morphology of 4T1 cells after different treatments. Scale bar, 200 nm. (**E** and **F**) Quantitative analysis of the number (E) and size (F) of membrane pores on 4T1 cells after various treatments. (**G**) Fluorescence microscopy images of FITC entry into 4T1 cells following treatments. Scale bar, 100 μm. (**H** and **I**) Flow cytometry analysis (H) and quantification (I) of FITC fluorescence in 4T1 cells. (**J**) Fluorescence recovery of 4T1 cells labeled with NBD-C6-HPC after photobleaching. Time points include prebleach, bleach, and recovery phases. Dashed circles indicate the bleached regions. Scale bars, 10 μm. (**K**) Normalized fluorescence intensity over time in the bleached regions. (**L** to **N**) The analysis of GZMA^FITC^ delivery via LipoNCs or electroporation: flow cytometry plots [Created in BioRender. Z. Zhang (2026); https://BioRender.com/u8kp77s] (L), mean fluorescence intensity (M), and positivity rates (N). (**O**) Cell viability after GZMA^FITC^ delivery via electroporation or LipoNCs. (**P**) Schematic illustrating the mechanism of enhanced intracellular GZMA delivery facilitated by LipoNC-induced membrane pore formation. (**Q**) Protein expression of full-length GSDMB and its cleaved p30 fragment in 4T1 cells after treatment with ^OPSA^LipoNCs@pGSDMB or ^OPSA^LipoNCs@pVector, with or without 10 μg of GZMA. (**R**) Bright-field microscopy images of 4T1 cells after treatment with ^OPSA^LipoNCs@pGSDMB or ^OPSA^LipoNCs@pVector with or without 10 μg of GZMA, highlighting pyroptotic body formation (yellow arrows). Scale bar, 50 μm. (**S**) Schematic illustrating GSDMB overexpression and cleavage by GZMA to initiate pyroptosis. Data are presented as means ± SD. Statistical comparisons among groups were performed using one-way ANOVA.

To improve in vivo delivery efficacy, we engineered fusogenic liposomal NCs (LipoNCs) by enveloping NCs with a lipid bilayer composed of 1,2-dioleoyl-*sn*-glycero-3-phosphoethanolamine (DOPE), cholesteryl hemisuccinate (CHEMS), and 1,2-distearoyl-*sn*-glycero-3-phosphoethanolamine *N*-[methoxy(polyethylene glycol)-2000] (DSPE-PEG) (Fig. 3A) (*37*). This formulation yielded LipoNCs with a hydrodynamic diameter of ~110 nm and a near-neutral ζ potential of −5 mV, as characterized by DLS and cryo-TEM (Fig. 3, B and C, and fig. S21). Nanoflow cytometry confirmed complete encapsulation of NCs within the lipid vesicles (fig. S22). The PEGylated surface would impart stealth properties for prolonged circulation, while the fusogenic lipid composition was intended to promote direct cytosolic delivery via membrane fusion (*37*).

To elucidate the internalization mechanism, we constructed dual-labeled LipoNCs using Cy5-labeled DNA (^Cy5^DNA) and DiI-labeled lipid membranes. Upon incubation with tumor cells, ^Cy5^DNA and DiI signals initially colocalized at the plasma membrane, consistent with early membrane interactions. Over time, ^Cy5^DNA signals redistributed into the cytosol and even nucleus, with minimal overlap with lysosomes, whereas DiI fluorescence remained primarily membrane-associated (fig. S23). Moreover, the cellular uptake of LipoNCs was markedly reduced at 4°C but largely unaffected by pharmacological inhibitors of clathrin-mediated endocytosis (chlorpromazine), caveolae-mediated endocytosis (filipin), macropinocytosis (wortmannin), or actin polymerization (cytochalasin D) (fig. S24). These results collectively support membrane fusion as the predominant route of cellular entry for LipoNCs.

The OPSA polymer within LipoNCs (^OPSA^LipoNCs) was designed to exploit the membrane-permeabilizing properties of phenylboronate-functionalized polycations, which transiently disrupt lipid bilayers and facilitate cytosolic delivery of macromolecules such as nucleic acids and proteins (*38*). Compared to PEI-based LipoNCs (^PEI^LipoNCs), ^OPSA^LipoNCs induced significantly more abundant and larger membrane pores, as visualized by scanning electron microscope (SEM) (Fig. 3, D to F). This enhanced pore-forming activity enabled the efficient cytosolic delivery of normally impermeable cargo, such as fluorescein isothiocyanate (FITC), which readily entered ^OPSA^LipoNC-treated cells but not controls (Fig. 3, G to I). Mechanistically, electrostatic and hydrophobic interactions between ^OPSA^LipoNCs and the inner cell membrane likely generate a “tug-of-war” effect (*39*), stretching and permeabilizing the lipid bilayer of cell membranes. Fluorescent recovery after photobleaching (FRAP) assays revealed that membrane fluidity was markedly reduced after ^OPSA^LipoNC treatment (Fig. 3, J and K), indicating compromised membrane integrity and delayed recovery dynamics. Crucially, this permeabilization was reversible, with pore resealing and FITC exclusion restored within 1 hour of ^OPSA^LipoNC removal (fig. S25). These findings demonstrate that ^OPSA^LipoNCs enable controlled, temporary access for therapeutic molecules without causing lasting cellular damage.

Inducing GSDMB-mediated pyroptosis requires efficient cytosolic delivery of GZMA to cleave GSDMB, a process often thwarted by the membrane repair mechanisms of tumor cells (*40*). To bypass this barrier, we leveraged the transient pore-forming capability of ^OPSA^LipoNCs, achieving intracellular GZMA delivery efficiencies comparable to electroporation (Fig. 3, L to N) while maintaining more than 90% cell viability (Fig. 3O). This perforation strategy facilitated efficient transmembrane transport of GZMA (Fig. 3P), circumventing the self-repair defenses. In GSDMB-overexpressing 4T1 cells, coincubation with GZMA and ^OPSA^LipoNCs drove robust GSDMB cleavage (fig. S26). Furthermore, treatment with pGSDMB-loaded ^OPSA^LipoNCs (^OPSA^LipoNCs@pGSDMB) synergistically enhanced GSDMB expression and pyroptosis induction, as evidenced by the cleavage of full-length GSDMB (Fig. 3Q) and the formation of pyroptotic bodies (Fig. 3R). The oligomerization of GSDMB-N into membrane pores (*36*) triggers cell lysis and the release of pro-inflammatory cytokines (Fig. 3S). These findings demonstrate that ^OPSA^LipoNCs effectively induce GSDMB-dependent pyroptosis through enhanced GZMA delivery and subsequent GSDMB cleavage, underscoring a promising strategy for eliciting tumor cell pyroptosis and bolstering antitumor immunity.

### LipoNCs@pGSDMB exhibit enhanced therapeutic efficacy against bone metastasis

**Fig. 4.**
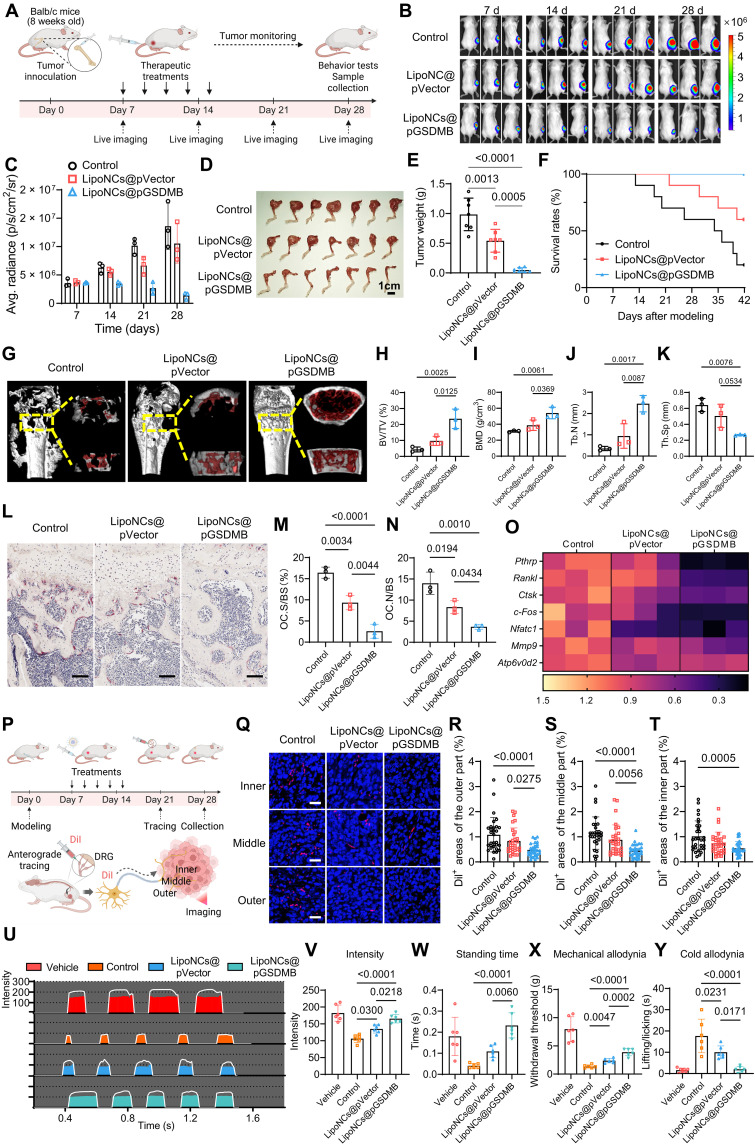
LipoNCs@pGSDMB demonstrate potent tumor-killing effects, neural modulation, and bone destruction prevention. (**A**) Schematic of in vivo experimental design. Created in BioRender. Z. Zhang (2026); https://BioRender.com/26558kg. (**B**) In vivo bioluminescence imaging of bone metastases following treatment (regimen as in Fig. 3A); *n* = 3. (**C**) Quantification of the averaged bioluminescence intensity over time for each group. (**D**) Images of tumor-bearing bones at the end point; *n* = 7. (**E**) Tumor weights across different groups. *n* = 7. (**F**) Survival curve of mice for each treatment group over 42 days. *n* = 10. (**G**) Micro-CT images of tumor-bearing legs. (**H** to **K**) Quantitation of bone microstructural parameters: bone volume fraction (BV/TV) (H), bone mineral density (BMD) (I), trabecular number (TB.N) (J), and trabecular separation (Th.Sp) (K). (**L** to **N**) Tartrate-resistant acid phosphatase (TRAP) staining images of distal femoral epiphysis (L), with quantification of osteoclast surface area (OC.S/BS) (M) and osteoclast number (OC.N/BS) (N); *n* = 3. Scale bars, 100 μm. (**O**) mRNA expression levels of *Pthrp* and *Rankl* in tumors and *Ctsk*, *c-Fos*, *Nfatc1*, *Mmp9*, and *Atp6v0d2* in the bone marrow of tumor-bearing legs. (**P**) Schematic of anterograde tracing of sensory nerves ipsilateral to the tumor. At week 3, the nerve tracer DiI was injected into the lumbar DRGs at the tumor site. At week 4, tumors were harvested for analysis. Created in BioRender. Z. Zhang (2026); https://BioRender.com/m6yjcdn. (**Q** to **T**) Fluorescent images of traced nerves in outer, middle, and inner tumor regions (Q) and quantification of DiI^+^ area in outer (R), middle (S), and inner (T) tumor regions; *n* = 3, with 10 independent regions of interest (ROIs) per sample. Scale bars, 20 μm. (**U** to **W**) CatWalk gait analysis of tumor-bearing legs (U), including contact intensity (V) and standing time (W). (**X** and **Y**) Pain assessment in tumor-bearing legs via Von Frey test (X) and acetone test (Y). *n* = 6. Data are presented as means ± SD. Statistical comparisons among groups were performed using one-way ANOVA.

We next assessed the therapeutic efficacy of LipoNCs@pGSDMB in a murine model of 4T1 breast cancer bone metastasis. To establish the model, 4T1 cells were directly injected into the femoral bone marrow cavity via a predrilled hole, enabling localized colonization within the bone microenvironment (Fig. 4A). One week postinoculation, intravenous administration of LipoNCs resulted in substantial accumulation within bone lesions, reaching fluorescence intensities comparable to those in the liver by 48 hours (fig. S27). Flow cytometric analysis further confirmed preferential uptake of LipoNCs by tumor cells within the bone microenvironment (fig. S28). Notably, intrafemoral metastatic bone tumors exhibited significantly higher ROS levels compared to subcutaneous tumors (fig. S29), which was even more pronounced in a more aggressive intra-iliac artery model (fig. S30), providing a favorable redox milieu for selective gene release and activation of LipoNCs@pGSDMB.

To assess treatment outcomes, we employed a luciferase-expressing 4T1 (4T1-Luc) bone metastasis model and monitored tumor progression using bioluminescence imaging following the regimen outlined in Fig. 4A. Strikingly, LipoNCs@pGSDMB induced a marked reduction in tumor burden, achieving near-complete eradication of bone lesions by day 28 (Fig. 4, B and C). At the experimental end point, femurs and tibias were harvested to quantify intraosseous tumor burden (Fig. 4D). While LipoNCs@pVector achieved a tumor inhibition rate of 51%, LipoNCs@pGSDMB led to an impressive inhibition rate of 94% (Fig. 4E). This therapeutic benefit translated into substantially extended survival, with 100% of mice in the LipoNCs@pGSDMB group surviving to day 42, compared to 60% in the LipoNCs@pVector group and only 20% in controls (Fig. 4F). To more closely mimic the clinical scenario of spontaneous bone metastasis, we established a physiologically relevant model via intra-iliac artery injection (fig. S31A). This model exhibited higher malignancy, characterized by accelerated tumor growth, earlier onset of mortality, and frequent multiorgan dissemination (fig. S31B). The LipoNCs@pGSDMB treatment remained highly effective, nearly eliminating metastatic lesions and preventing their spread beyond the bone (fig. S31, B to E). Histological (hematoxylin and eosin) and immunofluorescence analyses confirmed that LipoNCs@pGSDMB treatment induced robust tumor cell apoptosis and suppressed proliferation (fig. S32). LipoNCs@pGSDMB treatment did not cause notable changes in body weight (fig. S33), histopathological abnormalities in major organs (fig. S34), or hepatic dysfunction (fig. S35), supporting a favorable safety profile.

Bone metastases are frequently accompanied by severe skeletal degradation, driven either by acidic tumor secretions that erode the bone matrix or by enhanced osteoclast activity that accelerates bone resorption (*41*, *42*). Accordingly, effective tumor eradication is anticipated to confer protective effects on bone integrity. Micro–computed tomography (micro-CT) and three-dimensional (3D) reconstructions revealed that LipoNCs@pGSDMB treatment substantially preserved bone architecture in metastatic lesions (Fig. 4G). Quantitative analyses demonstrated significant improvements in bone volume fraction, bone mineral density, and trabecular number, alongside reduced trabecular separation (Fig. 4, H to K). Consistent with attenuated osteolysis, tartrate-resistant acid phosphatase (TRAP) staining showed decreased osteoclast activation in tumor-bearing femurs of LipoNCs@pGSDMB-treated mice (Fig. 4L), evidenced by decreased surface area and osteoclast number relative to bone surface (Fig. 4, M and N). Furthermore, quantitative polymerase chain reaction (qPCR) analyses corroborated the downregulation of key osteoclast-associated genes, including *Ctsk*, *c-Fos*, *Nfatc1*, *Mmp9*, and *Atp6v0d2* in bone marrow of the tumor-bearing limbs, as well as upstream regulators, *Rankl* and *Pthrp*, in tumors (Fig. 4O), which are critical in initiating osteoclast differentiation. These findings collectively indicate that LipoNCs@pGSDMB protect bone structural integrity by simultaneously eliminating metastatic tumor cells and suppressing osteoclast-mediated bone resorption.

Given the emerging recognition of sensory nerves as active participants in bone metastasis progression, we next evaluated whether LipoNCs@pGSDMB modulate tumor-nerve interactions in vivo. Previous studies have demonstrated that STING activation can attenuate sensory neuron excitability in bone cancer models (*43*), suggesting potential neuroregulatory benefits of our system in bone-metastatic settings. To visualize tumor-innervating sensory fibers, we performed anterograde axonal tracing by microinjecting the lipophilic dye DiI into L1-L4 dorsal root ganglia (DRGs), which innervate the mouse hindlimb, the site of tumor implantation in the intrafemoral metastasis model (Fig. 4P). While DRG neuronal somata reside within the spinal column, their axons extend peripherally to infiltrate tumor tissues and integrate into the local microenvironment. DiI-based fluorescence imaging revealed that LipoNCs@pGSDMB markedly reduced sensory axon infiltration across the outer, medial, and central tumor regions compared to LipoNCs@pVector and control groups (Fig. 4, Q to T).

To assess functional outcomes of tumor-nerve modulation, we conducted CatWalk gait analysis to evaluate motor coordination and limb weight-bearing in tumor-bearing mice (Fig. 4U). Mice treated with LipoNCs@pGSDMB exhibited improved limb endurance and weight distribution on the tumor-bearing hindlimb (Fig. 4, V and W), suggesting alleviation of nociceptive burden. Consistently, behavioral pain assays, including Von Frey filament testing and acetone-induced cold allodynia, revealed significantly reduced mechanical and cold hypersensitivity in the LipoNCs@pGSDMB group (Fig. 4, X and Y). Together, these findings demonstrate that LipoNCs@pGSDMB not only inhibit tumor growth and skeletal destruction but also mitigate aberrant nerve infiltration and nociceptive signaling, thereby offering dual benefits in tumor suppression and pain relief for bone metastasis.

### LipoNCs@pGSDMB elicit robust antitumor immunity in vivo

**Fig. 5.**
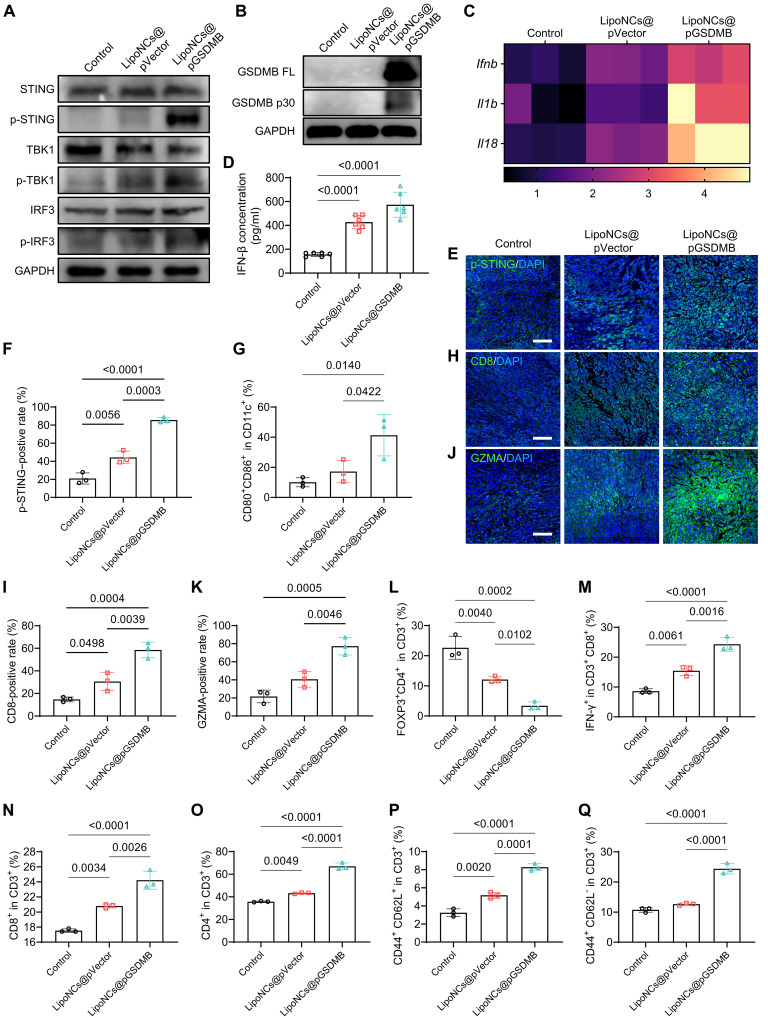
LipoNCs@pGSDMB enhance antitumor immune responses in vivo. (**A**) Western blot analysis of STING, TBK1, IRF3, and their phosphorylated forms in tumor tissues following various treatments. (**B**) Western blot analysis of full-length GSDMB and its cleaved p30 fragment in tumor tissues. (**C**) mRNA expression levels of *Ifnb*, *Il1b*, and *Il18* in tumor tissues. (**D**) Quantification of IFN-β levels in mouse serum. *n* = 6. (**E** and **F**) Immunofluorescence images (E) and quantification (F) of p-STING^+^ cells in tumor tissues. Green: p-STING, Blue: 4′,6-diamidino-2-phenylindole (DAPI). *n* = 3. Scale bar, 100 μm. (**G**) Flow cytometry analysis showing the proportion of mature DCs (CD11c^+^CD80^+^CD86^+^) in TDLNs. *n* = 3. (**H** and **I**) Immunofluorescence images (H) and quantification (I) of CD8^+^ T cells in tumor tissues. Green: CD8, Blue: DAPI. *n* = 3. Scale bar, 100 μm. (**J** and **K**) Immunofluorescence images (J) and quantification (K) of GZMA^+^ cells in tumor tissues. Green: GZMA, Blue: DAPI. *n* = 3. Scale bar, 100 μm. (**L** and **M**) Flow cytometry analysis showing the proportions of Treg cells (CD3^+^CD4^+^FOXP3^+^) (L) and activated CTLs (CD3^+^CD8^+^IFN-γ^+^) (M) in tumor tissues. *n* = 3. (**N** to **Q**) Flow cytometry analysis of the proportions of CTLs (CD3^+^CD8^+^) (N), Ths (CD3^+^CD4^+^) (O), Tcms (CD3^+^CD44^+^CD62L^+^) (P), and Tems (CD3^+^CD44^+^CD62L^−^) (Q) in spleens; *n* = 3. Data are presented as mean ± SD. Statistical comparisons among groups were performed using one-way ANOVA.

Following the potent therapeutic efficacy of LipoNCs@pGSDMB in eradicating bone metastases, we next sought to investigate the underlying mechanisms, with a particular emphasis on remodeling of the TIME (*44*, *45*). Mice bearing 4T1 bone metastases were treated according to the regimen shown in Fig. 4A. While LipoNCs@pVector triggered modest STING pathway activation in vivo, LipoNCs@pGSDMB robustly amplified antitumor immunity by concurrently activating STING signaling and inducing pyroptosis. Western blot analysis revealed pronounced phosphorylation of STING, TBK1, and IRF3 (Fig. 5A), alongside up-regulation and cleavage of GSDMB (Fig. 5B), confirming coactivation of innate immune and pyroptotic pathways. These molecular events were accompanied by elevated intratumoral levels of pro-inflammatory cytokines IFN-β, interleukin-1β (IL-1β), and IL-18 (Fig. 5C) and increased serum IFN-β (Fig. 5D), indicative of robust immune activation.

Further analysis revealed that LipoNCs@pGSDMB effectively remodeled the TIME within bone metastases. Immunofluorescence staining showed a marked increase in phosphorylated STING (p-STING) in tumors from LipoNCs@pGSDMB-treated mice, approximately twofold higher than in the LipoNCs@pVector group (Fig. 5, E and F). This was accompanied by significant enhancement of DC maturation in tumor-draining lymph nodes (TDLNs), as the proportion of CD80^+^CD86^+^ mature DCs increased from 10.0% in the control group to 17.2% in the LipoNCs@pVector group and further to 41.3% in the LipoNCs@pGSDMB group (Fig. 5G and fig. S36). Mature DCs promote antigen presentation and T cell priming (*46*), and correspondingly, intratumoral CD8^+^ CTL infiltration nearly doubled in the LipoNCs@pGSDMB group relative to LipoNCs@pVector (Fig. 5, H and I). Immunofluorescence analysis further revealed significantly elevated GZMA levels in LipoNCs@pGSDMB-treated tumors, representing a 1.9-fold increase over the LipoNCs@pVector-treated group (Fig. 5, J and K). The enhanced secretion of GZMA by CTLs is expected to drive tumor-specific pyroptosis via GSDMB cleavage, further amplifying antitumor immunity in a self-reinforcing manner.

In addition to augmenting effector T cell responses, LipoNCs@pGSDMB treatment significantly suppressed immunosuppressive components of the TME. Specifically, the proportion of T_reg_ cells (CD4^+^FOXP3^+^) within tumors was reduced by nearly 72% relative to LipoNCs@pVector-treated mice and to approximately one-seventh of untreated controls (Fig. 5L and fig. S37). This alleviation of local immune suppression was accompanied by a robust increase in activated IFN-γ^+^CD8^+^ T cells within tumors (Fig. 5M and fig. S38), indicating enhanced effector function and a shift from an immunologically “cold” to “hot” TIME, which is more conducive to antitumor responses.

To evaluate whether these immunological benefits extended systemically, we analyzed splenic lymphocyte populations. LipoNCs@pGSDMB treatment significantly increased the proportions of both CTLs (Fig. 5N and fig. S39) and CD4^+^ helper T cells (Ths) (Fig. 5O and fig. S40) while also stimulating the expansion of central memory T cells (Tcms) and effector memory T cells (Tems) (Fig. 5, P and Q, and fig. S41). These memory T cell subsets are critical for long-term immune surveillance and protection against tumor recurrence (*47*). Collectively, these results demonstrate that LipoNCs@pGSDMB reprogram both local and systemic immunity through a synergistic mechanism involving STING pathway activation and GSDMB-driven pyroptosis. By simultaneously reducing immunosuppressive Treg cells, enhancing effector T cell infiltration and function, and establishing durable immune memory, LipoNCs@pGSDMB represent a potent immunotherapeutic strategy for treating metastatic bone tumors.

### Calcium circuitry reprogramming disrupts tumor-nerve cross-talk

**Fig. 6.**
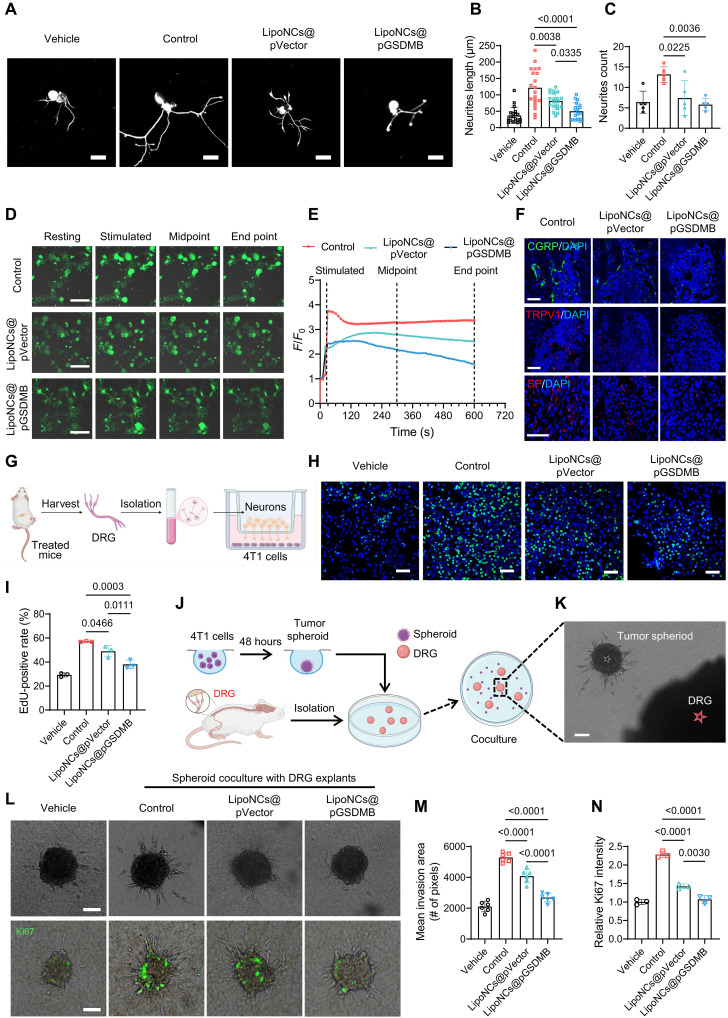
LipoNCs target the tumor-nerve axis to inhibit tumor metastasis. (**A**) Immunofluorescence images of Tuj1 protein in DRG neurons cultured for 24 hours, isolated at day 28 posttreatment as described in Fig. 4A. Scale bars, 50 μm. (**B** and **C**) Quantification of neurite length (B) and branching numbers (C) in DRG cells across groups. (**D**) Time-lapse calcium imaging in DRG neurons isolated from tumor-bearing mice following various treatments. Scale bars, 100 μm. (**E**) Normalized fluorescence intensity changes during calcium imaging, calculated as *F*/*F*_0_ (*F*: instantaneous fluorescence intensity; *F*_0_: baseline fluorescence intensity). (**F**) Immunofluorescence images of pain-related markers (CGRP, TRPV1, and SP) in tumor-bearing legs across groups. Scale bars, 50 μm. (**G** to **I**) Effects of DRG neurons on tumor proliferation: schematic of tumor-nerve coculture in the Transwell device [Created in BioRender. J. Xiang (2026); https://BioRender.com/wx2os5x] (G), representative fluorescence images of EdU-stained 4T1 tumor cells cocultured with DRG neurons from various treatment groups (H), and quantitative analysis of EdU-positive rates of 4T1 cells. *n* = 3 (I). Scale bar, 100 μm. (**J** to **N**) The effects of DRG on the tumor invasion and proliferation; schematic of spheroid-DRG coculture setup [Created in BioRender. Z. Zhang (2026); https://BioRender.com/u8kp77s] (J), bright-field image of the coculture system (K), images of tumor spheroid invasion (12 hours) and Ki67 proliferation (24 hours) (L), and quantification of spheroid invasion area (*n* = 6) (M) and Ki67 fluorescence intensity (*n* = 3) (N). Scale bars, 50 μm.

To elucidate how LipoNCs@pGSDMB modulate sensory nerve outgrowth and excitability and subsequently influence tumor progression, we first evaluated their effects on DRG neurons. DRG neurons isolated from tumor-bearing mice exhibited pronounced neurite lengthening and branching in response to tumor stimulation, indicative of enhanced neurogenesis and nerve infiltration into the TME. Treatment with either LipoNCs@pVector or LipoNCs@pGSDMB significantly suppressed this tumor-induced neurogenesis, with LipoNCs@pGSDMB showing the greatest reduction in neurite outgrowth (Fig. 6, A to C). To assess whether LipoNCs@pGSDMB also influence neuronal excitability, we measured calcium flux (*48*) in ex vivo DRG tissues following depolarization with potassium chloride (KCl), which triggers action potential firing and mimics nociceptive stimulation (*49*). Fluo-4 AM, a calcium-sensitive dye, was used to track intracellular calcium dynamics. DRG neurons from LipoNCs@pGSDMB-treated mice displayed markedly attenuated calcium responses, characterized by lower calcium flux amplitude and faster recovery to resting levels, compared to neurons from control or LipoNCs@pVector-treated mice (Fig. 6, D and E), indicating reduced neuronal excitability.

We next explored the downstream consequences of decreased neuronal excitability by examining key neuropathic sensitization markers, including CGRP (*50*), transient receptor potential vanilloid 1 (TRPV1) (*51*), and substance P (SP) (*52*), in tumor-bearing bones. Immunofluorescence staining revealed substantial reductions in these neuroinflammatory mediators in mice treated with LipoNCs@pGSDMB (Fig. 6F). Given that CGRP, TRPV1, and SP are strongly implicated in driving neuroinflammation and neuropathic pain, both of which can accelerate tumor progression and bone destruction in metastases, their suppression implies a dual benefit of LipoNCs@pGSDMB: alleviating cancer-associated pain and disrupting the neuroinflammatory niche that fosters metastatic progression.

To directly assess the influence of tumor-nerve interactions on tumor progression, we used a Transwell coculture system with DRG neurons in the upper compartment and 4T1 tumor cells in the lower compartment (Fig. 6G). DRG neurons from untreated tumor-bearing mice significantly enhanced tumor cell proliferation, whereas those from LipoNCs@pGSDMB-treated mice strongly suppressed this trend, as evidenced by reduced 5-ethynyl-2′-deoxyuridine (EdU) incorporation (Fig. 6, H and I). In addition, a 3D coculture model was established by embedding DRG explants from each treatment group into collagen I gels, along with 4T1 tumor spheroids (Fig. 6, J and K). Tumor spheroids cocultured with untreated DRGs exhibited enhanced invasion and proliferation, reflecting the prometastatic role of tumor-innervating nerves. By contrast, spheroids cultured with DRGs from LipoNC-treated mice, particularly those receiving LipoNCs@pGSDMB, resulted in a significant reduction in both phenotypes (Fig. 6, L to N). These observations suggest that LipoNCs@pGSDMB effectively dismantle the tumor-nerve cross-talk, thereby limiting the proproliferative and proinvasive cues derived from sensory neurons.

**Fig. 7.**
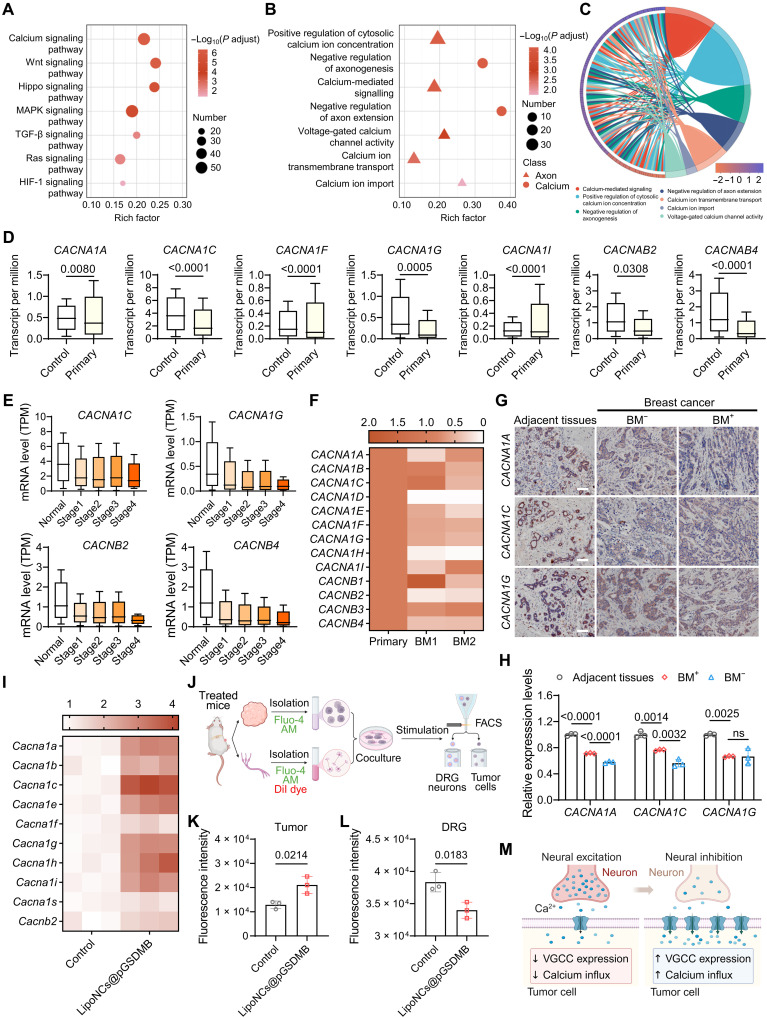
LipoNCs modulate the tumor-nerve cross-talk via calcium signaling. (**A**) KEGG enrichment analysis comparing the LipoNCs@pGSDMB and control groups. (**B**) GO enrichment analysis comparing the same groups. (**C**) Chord diagram illustrating the enrichment of pathways related to calcium ion influx and axon negative regulation. The scale reflects the log_2_ fold change (log_2_FC) of gene expression, with the pathways depicted in the diagram accordingly. (**D**) VGCC expression profiles in normal tissues (*n* = 114) and primary breast cancer tissues (*n* = 1097) were analyzed using the TCGA data in the UALCAN database. (**E**) Expression levels of calcium channel–related genes across different stages of breast cancer obtained from the ULCAN database. TPM, transcripts per million. (**F**) Expression profiles of VGCCs in primary breast cancer and bone metastases (BM1 and BM2) derived from GSE190772. (**G**) Immunohistochemistry images of VGCCs in adjacent normal tissues, breast cancer tissues from patients without bone metastases (BM^−^), and those with bone metastases (BM^+^). Scale bars, 100 μm. (**H**) Quantitative analysis of VGCC expression in adjacent normal tissues and breast cancer tissues without (BM^−^) or with (BM^+^) future bone metastasis. (**I**) VGCC expression levels in tumor cells analyzed by qPCR. Mice were treated with PBS or LipoNCs@pGSDMB according to the regimen in Fig. 4A. (**J** to **L**) Schematic illustration of calcium flux assays in cocultured primary tumor cells and DRG neurons [Created in BioRender. Z. Zhang (2026); https://BioRender.com/jm9a50l] (J) and quantitative fluorescence intensity of calcium probes under KCl stimulation in cocultured tumor cells (K) and DRG neurons (L). (**M**) Schematic representation of the modulation of VGCCs and calcium influx by LipoNCs@pGSDMB. Data are presented as means ± SD. Statistical analyses involving two groups were conducted using Student’s *t* test, while comparisons among more than two groups were carried out using one-way ANOVA. Created in BioRender. Z. Zhang (2026); https://BioRender.com/ztlbmfo.

To uncover the molecular underpinnings of these effects, we performed RNA sequencing on 4T1-GFP cells isolated from bone metastases by fluorescence-activated cell sorting (FACS). Differential expression analysis revealed notable transcriptional reprogramming in pathways involved in immune regulation and cell signaling (figs. S42 and S43). Kyoto Encyclopedia of Genes and Genomes (KEGG) pathway analysis pointed to a marked enrichment of calcium signaling (Fig. 7A), which matters in both tumor biology and neuronal function (*53*, *54*). In addition, Wnt signaling, which regulates calcium homeostasis (*55*), was also enriched. Gene Ontology (GO) analysis further highlighted terms associated with calcium ion influx and negative regulation of axon extension (Fig. 7B), with most related genes being markedly up-regulated (Fig. 7C). Notably, several semaphorin (Sema) family members and Slit1, known to inhibit axon outgrowth through cytoskeletal reorganization and growth cone collapse (*56*, *57*), were substantially elevated (fig. S44), potentially accounting for the observed reduction in tumor-infiltrating nerves in LipoNCs@pGSDMB-treated mice.

Among the most prominently altered genes in 4T1 tumor cells isolated from LipoNCs@pGSDMB-treated mice were those encoding VGCCs (fig. S45), key regulators of calcium-dependent processes including tumor proliferation, invasion, and metastasis (*58*). Although tumor cells are not classically excitable, they do express VGCCs that permit calcium influx upon depolarization (*59*). Mining The Cancer Genome Atlas (TCGA) data via UALCAN (*60*, *61*) revealed that VGCC expression is generally downregulated in tumor tissues (Fig. 7D), with even lower levels associated with advanced stages and metastatic burden (Fig. 7E). Single-cell omics data from breast cancer patients with bone metastases to the pelvis and tibia (*62*) further corroborated a marked reduction in VGCC-related genes in bone metastases (BM1 and BM2) compared to primary tumors (Fig. 7F). The immunohistochemical staining of clinical tumor specimens also confirmed markedly diminished VGCC expression in patients who later developed bone metastases (BM^+^) (Fig. 7, G and H). Elevated VGCC levels correlated with prolonged overall survival in patients with breast cancer according to Kaplan-Meier Plotter database analysis (hazard ratio < 1, *P* < 0.05) (fig. S46, A and B) (*63*). Moreover, in chemotherapy-treated cohorts (*64*), responders exhibited 1.4-fold higher VGCC expression than nonresponders (fig. S46C), with receiver operating characteristic (ROC) analysis further validating VGCCs as predictive biomarkers of treatment sensitivity in breast cancer (fig. S46D). Tumor cells from LipoNCs@pGSDMB-treated mice regained VGCC expression, as confirmed by qPCR (Fig. 7I) and Western blot analyses (fig. S47).

To assess the functional consequences of VGCC reactivation, we monitored calcium flux using Fluo-4 AM–labeled tumor cells isolated from treated mice. While basal calcium levels were unchanged, KCl stimulation elicited significantly enhanced calcium influx in tumor cells from the LipoNCs@pGSDMB group compared to controls (fig. S48). To investigate the implications of these changes for tumor-nerve cross-talk, we established a coculture model using DiI-labeled DRG neurons and 4T1 tumor cells harvested from treated mice (Fig. 7J). Notably, KCl-evoked calcium influx was markedly augmented in tumor cells from LipoNCs@pGSDMB-treated mice, while neuronal calcium flux was diminished in the same cocultures (Fig. 7, K and L). This inverse calcium signaling pattern suggested a competitive dynamic between tumor and nerve cells. Transient membrane perforation by LipoNCs does not explain these observations, as direct exposure of 4T1 cells to LipoNCs failed to alter baseline calcium levels (fig. S49). Instead, we propose that VGCC up-regulation in tumor cells enhances their calcium uptake capacity, effectively sequestering extracellular calcium and depriving adjacent neurons of calcium availability (Fig. 7M). Given the critical role of calcium in neuronal processes, from neural progenitor proliferation to synaptic function (*65*, *66*), calcium depletion is likely to constrain neurogenesis and excitability, thereby impairing tumor innervation (*67*). Together, these findings demonstrate that LipoNCs@pGSDMB reprogram calcium circuitry in tumor cells, disrupting tumor-nerve cross-talk and thus mitigating both metastatic progression and cancer-associated pain.

## DISCUSSION

Bone metastasis arises when disseminated tumor cells evade immune surveillance and colonize the bone marrow niche, a microenvironment characterized by profound immunosuppression and intricate tumor-nerve cross-talk. Conventional therapies often overlook these interwoven pathologies, leading to poor tumor control and persistent neuropathic pain driven by aberrant neuronal activation (*68*). Here, we report a dual-targeting nanotherapy, LipoNCs@pGSDMB, that concurrently dismantles immunosuppressive barriers and disrupts tumor-nerve interactions, establishing a feasible paradigm for bone metastasis treatment.

STING agonists hold promise for immune activation (*69*) and neuronal modulation (*43*), yet their clinical utility is limited by rapid systemic clearance, off-target effects, and resistance mechanisms (*23*, *70*). To address these challenges, we developed a tumor-activated platform codelivering the OPSA prodrug and pGSDMB using fusogenic liposomes. The ROS-rich bone TME enabled spatiotemporal control of payload release. OPSA promoted robust STING activation, evidenced by increased phosphorylation of STING, TBK1, and IRF3, as well as enhanced DC maturation, CTL infiltration, and GZMA secretion. LipoNCs@pGSDMB facilitated the cellular entry of GZMA through reversible membrane perforation, thereby triggering GSDMB-mediated pyroptosis. This immunogenic cell death (*36*), in turn, released tumor-associated antigens, further amplifying STING-driven immune priming. Simultaneously, LipoNCs@pGSDMB suppressed DRG excitability, reduced intratumoral axonogenesis, and dampened neural signals that otherwise drive tumor growth and invasion. This dual immunomodulatory and neuroregulatory synergy achieved 94% tumor inhibition and pain resolution in preclinical bone metastasis models.

Our multiomics analyses revealed that LipoNCs@pGSDMB reprogrammed calcium homeostasis in the tumor-nerve axis by restoring VGCC expression in metastatic tumor cells, a process often impaired in advanced malignancies. This restoration facilitated calcium influx into tumor cells, depleting the extracellular calcium pool available to adjacent neurons and thereby suppressing neuronal excitability. This calcium circuit remodeling may underlie the decreased expression of neuropathic markers (CGRP, TRPV1, and SP) and reduced tumor invasiveness. While VGCCs emerge as a promising therapeutic target, further investigation is required to delineate the downstream molecular pathways linking calcium signaling to neural and tumoral behaviors. In parallel, the potent STING activation induced by LipoNCs@pGSDMB elevated IFN-β levels both locally and systemically (*71*), which may further suppress sensory neuron excitability, thereby complementing VGCC-mediated calcium redistribution in disrupting tumor-nerve cross-talk.

Despite these advances, the broader applicability of this approach across diverse tumor types remains to be fully elucidated. Tumor-nerve cross-talk is highly context dependent, with considerable heterogeneity in neural composition, innervation density, and neurochemical signaling across malignancies such as pancreatic, prostate, gastric, and brain cancers (*72*). Whether VGCC modulation constitutes a conserved mechanism underlying tumor-nerve interactions or necessitates tumor-specific strategies remains an open question. Furthermore, while our system demonstrated promising safety and efficacy in preclinical models, the optimization of biodistribution and the long-term safety, particularly concerning the reversibility and tolerability of transient membrane perturbation, requires further evaluation.

In summary, we present a neuroimmunomodulatory nanotherapy that synergistically integrates STING activation, pyroptosis induction, and calcium circuit reprogramming to combat bone metastasis. By dismantling the immunosuppressive niche and decoupling tumor-nerve symbiosis, LipoNCs@pGSDMB achieved robust antitumor efficacy and effective pain relief. This work uncovers a previously underappreciated role for VGCCs in modulating tumor-nerve interactions, positioning them as actionable targets in innervated tumors. This work not only establishes a mechanistic foundation for precision neuroimmunotherapies but also underscores the translational potential of leveraging clinically available VGCC-targeting agents to enhance immune responses and mitigate cancer-associated pain.

## MATERIALS AND METHODS

### Cell culture

4T1, 4T1-Luc, 4T1-GFP, and LLC cells were cultured in Dulbecco’s modified Eagle’s medium (DMEM) supplemented with 10% fetal bovine serum (FBS), penicillin (100 U/ml), and streptomycin (100 μg/ml). Primary DRG neurons were isolated from the L1-L4 spinal segments of mice using the previously described method (*73*). Following euthanasia via carbon dioxide asphyxiation, the spines were dissected and separated longitudinally. DRGs innervating the tumor region were carefully excised. DRG tissues were digested in type II collagenase (3 mg/ml) for 1 hour at 37°C, followed by an additional 10-min digestion with 0.1% trypsin. FBS was added to terminate enzymatic activity. The digested tissues were gently triturated with a 1-ml syringe needle and filtered through a 40-μm cell strainer to generate a single-cell suspension. The suspension was centrifuged, and the resulting pellet was resuspended in a Neurobasal medium containing 2% B27. DRG neurons were seeded on dishes precoated with poly-l-lysine and cultured under standard conditions.

### In vitro and in vivo STING activation

To evaluate STING pathway activation in vitro, 4T1 or LLC cells (3 × 10^5^) were transfected with NCs@pVector or NCs@pGSDMB in six-well plates. Transfection was performed in serum-free DMEM for 4 hours, followed by replacement with complete DMEM and a 48-hour incubation. Cells and culture supernatants were subsequently collected for analysis. Cell lysates were analyzed using western blotting to detect STING pathway activation markers, including STING, p-STING, IRF3, p-IRF3, TBK1, and p-TBK1. Culture supernatants were analyzed for cytokine release using Mouse IFN-β ELISA Kits. For oxidative stress conditions, following the 4-hour transfection, the medium was exchanged for complete DMEM supplemented with H_2_O_2_ at final concentrations of 0, 10, 50, 100, or 200 μM, followed by 48 hours of incubation. Cells and supernatants were then processed as described. For in vivo analysis, tumor tissue lysates were prepared from harvested tumors using the same protocol, and mouse serum samples were collected for cytokine quantification.

### In vitro and in vivo pyroptosis induction

For in vitro pyroptosis induction, 4T1 or LLC cells were first transfected with NCs@pVector or NCs@pGSDMB in six-well plates as described above. After 48 hours, cells were trypsinized, centrifuged, and resuspended in phosphate-buffered saline (PBS) at ~1 × 10^7^ cells/ml. The cell suspension was supplemented with 10 μg of GZMA protein and then transferred to electroporation cuvettes. Electroporation was conducted with parameters set to 300 V, 20 ms, and 2 pulses, enabling GZMA delivery into cells to simulate CTL activity. Postelectroporation, the cells were seeded in six-well plates with a complete medium for a 6-hour incubation. Subsequently, cells were treated with the annexin V-FITC Apoptosis Detection Kit to visualize pyroptotic bodies via confocal microscopy. For the immune cell–induced pyroptosis, 4T1 cells were transfected as described above and cocultured with NK92 cells at varying effector-to-target ratios (0, 1:1, 2:1) for 24 hours. In addition, cells were collected for protein extraction to evaluate GSDMB cleavage via Western blot, while culture supernatants were analyzed for LDH and ATP levels. For in vivo analysis, tumor tissues were harvested from treated mice, lysed, and analyzed by Western blot analysis using the same protocol.

### Tumor cell invasion assessment

To evaluate the invasive potential of tumor cells surviving simulated pyroptosis, 4T1 cells were collected and resuspended in serum-free DMEM to mimic a starvation state. Pretreated 4T1 cells (3 × 10^4^) were plated in the apical compartment of the Transwell insert coated with 10% Corning Matrigel to simulate the extracellular matrix surrounding tumors. The basal compartment was supplemented with 500 μl of 10% FBS DMEM to serve as a chemotactic agent. After incubation for 48 hours, the inserts were removed and washed twice with PBS. The inserts were then fixed in 4% paraformaldehyde (PFA) for 30 min and rinsed twice with PBS. Noninvasive cells and remaining Matrigel were gently wiped from the upper surface of the inserts using a cotton swab. Fixed cells on the underside of the inserts were stained with a 0.1% crystal violet solution for 15 min, followed by two PBS washes. Inserts were air-dried at 37°C before imaging under a microscope using a 10× objective lens. Crystal violet was solubilized in ethanol, and its absorbance at 595 nm was measured using a spectrophotometer to quantify cell invasion.

### Cell membrane perforation induced by LipoNCs

SEM was first performed to observe the cell surface pores. 4T1 cells (1 × 10^5^) were plated onto coverslips in six-well plates and incubated for 24 hours. ^OPSA^LipoNCs or ^PEI^LipoNCs (N/P = 15 for OPSA and 7 for PEI, lipid/mass ratio = 240, total plasmid of 4 μg) were then added for 8-hour incubation. Cells were rinsed thrice with PBS and fixed overnight in a 2.5% glutaraldehyde solution at 4°C. After three additional PBS washes, cell samples were treated with 1% osmium tetroxide at room temperature for 2 hours, followed by another three PBS washes. The fixed cells were dehydrated using ethanol solutions, dried in a Hitachi HCP-2 critical point dryer, and coated with gold. The processed samples were imaged using a Zeiss G300 SEM. For quantitative analysis, a fixed region of interest (ROI) measuring 1 μm^2^ was defined in each micrograph. Pores within the ROI were manually counted, and their diameters were measured using ImageJ software. A total of 20 individual cells were analyzed, and the resulting data were compiled for statistical analysis.

The hydrophilic and cell-impermeable FITC dye was used to assess the cell membrane perforation after treatments. 4T1 cells (1 × 10^5^) were incubated in six-well plates for 24 hours. ^OPSA^LipoNCs or ^PEI^LipoNCs containing the blank pVector were added at the same dosages as described above. Following 8 hours of incubation, each well was treated with 1 μg of FITC and incubated for 1 hour. Following three washes with PBS, cells were either directly observed via confocal microscopy or trypsinized for flow cytometry analysis of FITC fluorescence intensity. The presence of intracellular FITC, which can only enter through perforations, served as an indicator of membrane pore formation.

### FRAP to assess membrane fluidity

4T1 cells (5 × 10^3^) were incubated in confocal dishes for 24 hours. Then, cells were treated with either ^PEI^LipoNCs@pVector or ^OPSA^LipoNCs@pVector containing 1 μg of blank plasmid. After 8 hours, the medium was replaced with PBS, and cells were stained with the cell membrane dye NBD-C6-HPC (1:1000) for 15 min. After three washes with PBS, the solution was replaced with complete DMEM, and FRAP experiments were conducted using a Zeiss LSM880 confocal microscope. A selected portion of the cell membrane within the field of view was photobleached using a 488-nm wavelength laser at 100% power (25 mW). Fluorescence images were captured continuously throughout the bleaching process and subsequent recovery phase, with one image acquired every second. The data were analyzed using the FRAP Profiler V2 plugin in ImageJ to evaluate membrane fluidity based on the rate of fluorescence recovery in the bleached area.

### Intracellular GZMA delivery by LipoNCs

To compare the GZMA delivery efficiency between electroporation and LipoNCs, GZMA was labeled with FITC. 4 T1 cells (1 × 10^5^) were incubated in six-well plates for 24 hours. For the LipoNC-based delivery, cells were treated with ^OPSA^LipoNCs@pVector containing 4 μg of plasmid for 8 hours, followed by the addition of 10 μg of ^FITC^GZMA for an additional 1 hour of co-incubation. For electroporation-based delivery, cells were electroporated at 300 mV along with 10 μg of ^FITC^GZMA. The delivery efficiency was assessed by measuring intracellular fluorescence intensity and proportions of FITC-positive cells using flow cytometry. Cell viabilities were evaluated using the CCK-8 assay. For pyroptosis induction, GSDMB-overexpressing 4 T1 cells were treated with ^PEI^LipoNCs@pVector or ^OPSA^LipoNCs@pVector containing 1 μg of blank plasmid in combination with 10 μg of GZMA for 8 hours. In another experiment, 4 T1 cells were treated with ^OPSA^LipoNCs@pVector or ^OPSA^LipoNCs@pGSDMB containing 1 μg of plasmid, with or without 10 μg of GZMA, for 8 hours of co-incubation. After the treatments, cells were harvested for protein extraction and analyzed using Western blot.

### Animal and human use statement

All animal protocols were approved by the Institutional Animal Care and Use Committee (IACUC) of Zhejiang University and conducted in accordance with the guidelines of the Zhejiang University Animal Experimentation Committee (approval number ZJU20240278). Balb/c mice (18 to 20 g, female, 8 weeks old) were obtained from the Experimental Animal Center of the Zhejiang Academy of Medical Sciences and maintained under standard housing conditions. Surgically resected human breast cancer and adjacent normal tissues were from patients with breast cancer, with or without bone metastases, following a protocol approved by the ethics committee of Sir Run Run Shaw Hospital of Zhejiang University School of Medicine (approval number 2024-0442), with the informed consent of all patients.

### Establishment of bone metastasis model

An intrabone tumor model was established to simulate breast cancer bone metastasis. Briefly, Balb/c mice were anesthetized with sodium pentobarbital, and an incision was made at the knee joint to expose the patellar ligament. Using a micro-drill, a small hole was made in the femoral bone marrow cavity from the intercondylar fossa along the long axis of the femur. A micro-syringe was used to administer 5 μl of 4T1-Luc cell suspensions (1 × 10^5^ cells) into the bone marrow cavity. One week after tumor cell injection, luciferase substrate (150 mg/kg) was administered intraperitoneally. The mice were anesthetized using isoflurane, and bioluminescence imaging was performed to assess the successful establishment of bone metastasis.

For the intra-iliac artery bone metastasis model, BALB/c mice were anesthetized. After depilation of the inner thigh, a small skin incision was made with a scalpel to expose the underlying muscle. The femoral artery was then carefully isolated using blunt dissection. Under a stereomicroscope, 1 × 10^5^ 4T1-Luc cells suspended in 100 μl of PBS were injected into the iliac artery using a 31-gauge insulin syringe. Hemostasis was achieved by applying sterile gauze for 10 min, followed by skin closure with sutures. Mice were then placed on a heating pad for recovery. One week after tumor cell inoculation, luciferase substrate (150 mg/kg) was administered intraperitoneally. Bioluminescence imaging was performed to monitor the progression of bone metastasis.

### In vivo biodistribution study

To access the in vivo biodistribution, DiR-labeled LipoNCs (^DiR^LipoNCs) were prepared following the same preparation method as for LipoNCs. Two weeks after establishing the bone metastasis model, ^DiR^LipoNCs were administered via tail vein injection to the mice. At 8- and 48-hour postinjection, the mice were euthanized using carbon dioxide asphyxiation. Organs, including the heart, liver, spleen, lungs, kidneys, and tumor-bearing legs, were harvested and imaged to assess the distribution of ^DiR^LipoNCs. All imaging data were analyzed using Living Image 4 software for quantitative evaluation of fluorescence intensity.

### In vivo antitumor study

Mice bearing bone metastasis (both intrafemoral and intra-iliac artery model) were randomly assigned to three treatment groups: Control, LipoNCs@pVector, and LipoNCs@pGSDMB (*n* = 10). Treatment was initiated 1 week postmodeling, with intravenous injections of DNA (1 mg/kg) every other day for five injections. In vivo bioluminescence imaging was conducted weekly to track tumor progression in a subset of mice (*n* = 3), while tumor burden was quantified in the remaining animals. The mice body weight was measured every other day throughout the study. At the end point, behavioral tests were conducted to assess overall health and tumor burden. Following the tests, mice were euthanized via carbon dioxide asphyxiation, and tissues—including blood, heart, liver, spleen, lungs, kidneys, lymph nodes, tumors, and bone marrow—were harvested for further analysis. In survival experiments, tumor-bearing mice were grouped (*n* = 10) and treated under the same conditions as described above. Mice were euthanized upon reaching a tumor maximum diameter of 10 mm or experiencing more than 10% body weight loss.

### Anterograde tracing

Anterograde axonal tracing was performed by microinjecting DiI into the DRG one week before the 4-week end point. Tumor-bearing mice (*n* = 3) were anesthetized and positioned prone. After shaving the dorsal surface, a midline skin incision was made above the posterior superior iliac crest to expose the underlying musculature. A small muscle incision was created adjacent to the spinous process on the tumor-bearing side to access the vertebral column. The target DRG, located beneath the facet joint, was exposed by carefully excising the joint process with ophthalmic scissors. Following osteotomy, the accessory process was exposed, and the DRG was identified within its axilla. A total of 1 μl of DiI (1 mg/ml) was slowly injected into each DRG (L1-L4) using a microsyringe. The incision was then closed and sutured. At the 4-week endpoint, tumors were excised, embedded, and cryosectioned for fluorescence imaging. For quantification, the radial distance from the tumor margin to the center was divided into three concentric regions: outer, middle, and inner. In each region, ten representative ROIs (200 μm by 200 μm) were randomly selected per mouse for quantification.

### Behavior tests

At the 4-week end point, CatWalk, Von Frey, and acetone tests were conducted to evaluate pain responses in each group of mice. Six mice per group were tested, and data from each experiment were collected and analyzed for all behavioral assessments.

#### CatWalk test

Gait analysis was performed using an automated system (Noldus Information Technology B.V., Netherlands) to assess the mice’s pain response. The dynamic parameters of each paw, including contact time, pressure, and other relevant measures, were collected. All parameters were calculated by the analysis software (CatWalk XT 10.6).

#### Von Frey test

The Von Frey needle prick test was used to evaluate mechanical pain sensitivity. Mice were placed on a tactile testing platform consisting of a wire rack with fixed holes and an animal chamber. After a 30-min acclimation period, vertical stimulation was applied to the tumor-bearing paw using a Von Frey probe with a sensor. A positive response was recorded when the mouse retracted its paw, and the sensor automatically recorded the peak force. The threshold for a single response was defined as the force at which the mouse withdrew its paw. Each mouse underwent three trials, and the mean value was used as the threshold.

#### Acetone test

The acetone test was used to assess temperature-induced pain sensitivity. Mice were placed on the same tactile platform and allowed to acclimate. Acetone was then evenly applied to the tumor-bearing paw, where its rapid evaporation triggered a cold stimulus. Mice responded by licking or lifting the paw, and the duration of these responses was recorded to evaluate their sensitivity to cold pain.

### Micro-CT analysis

Intact femur and tibia bones were isolated by carefully removing the surrounding skin, muscle, and tumor tissues. The bones were then fixed in 4% PFA for 24 hours and rinsed twice with PBS. After fixation, the bones were positioned upright and scanned using a Micro-CT scanner (Skyscan 1275, Aartselaar, Belgium) with the following settings: 60-μA current, 50-kV voltage, and a resolution of 9 μm.

### Calcium flux assay

Flow cytometric analysis was performed to assess calcium flux in both primary tumor cells and DRG neurons. For tumor cells, cell suspensions were labeled with the Fluo-4 AM probe in calcium-containing Hanks’ balanced salt solution, and calcium flux was measured with or without KCl stimulation. To examine competitive calcium flux between tumor cells and neurons, tumor cells and DRG neurons were cocultured. DRG neurons were stained with DiI (5 μM) to distinguish them from tumor cells. Both cell types were then labeled with Fluo-4 AM and cocultured in calcium-containing HBSS at a tumor-to-neuron ratio of ~20:1. Calcium flux was measured under KCl stimulation, with the flux intensities for both tumor cells and neurons recorded separately.

### Calcium imaging

Calcium imaging was performed to assess the excitability of DRG neurons. After culturing the neurons at 37°C for 48 hours, they were stained with the Fluo-4 AM Calcium Assay Kit. Following three washes with PBS, the medium was replaced with a fresh Neurobasal medium, and continuous imaging was initiated. To simulate nonspecific depolarizing stimuli, the culture medium was supplemented with a final concentration of 30 mM KCl, which induced action potentials in the neurons. Calcium imaging and intensity measurements were performed with a Zeiss LSM 910 confocal microscope, with images captured every 5 s.

### Nerve-tumor cell Transwell coculture

A Transwell device was used to assess the ability of DRG neurons to promote tumor proliferation. Specifically, 1 × 10^4^ isolated DRG neurons from each group were seeded in the apical compartment of a 12-well Transwell insert, while 5 × 10^4^ 4T1 cells were plated in the basal compartment on coverslips. After 48 hours of coculture, tumor cell proliferation was evaluated using the EdU Cell Proliferation Kit. Following a 2-hour incubation with EdU, the cells were imaged using a confocal microscope. The proportion of EdU-positive cells was quantified using ImageJ software.

### Tumor spheroid-DRG invasion and proliferation assay

DRG explants were isolated from the L1-L4 segments on the tumor-bearing side of mice and seeded into 100% Corning Matrigel on a substrate precoated with rat tail collagen type I (1.5 mg/ml). Mature 4T1 spheroids were suspended in a rat tail collagen type I solution (1.5 mg/ml) and added to the culture dish containing the embedded DRG explants once the Matrigel solidified, maintaining a spheroid-to-DRG explant ratio of 5:1. After 12 hours of coculture, the tumor spheroids were imaged using a microscope. An overview of the spheroid-DRG coculture was captured using a 4× objective lens, while the invasion of individual spheroids was documented with a 10× objective lens. For the proliferation assay, an independent experiment was conducted, and the coculture was extended to 24 hours under the same conditions. Subsequently, the collagen matrix encapsulating the spheroids was rinsed with PBS, fixed in 4% PFA for 1 hour, and permeabilized with 0.1% Triton X-100 for another hour at room temperature. The samples were then blocked with blocking solution (Beyotime) for 1 hour and incubated overnight at 4°C with the Ki67 primary antibody (Proteintech). The following day, after thorough PBS washes, a fluorescent secondary antibody was applied for 2 hours at room temperature. The samples were then examined under a CLSM to assess tumor proliferation. The invasion area and Ki-67 fluorescence intensity were analyzed using ImageJ.

### Statistical analysis

Statistical analyses were conducted using GraphPad Prism (Version 9.5.0). For comparisons between two groups, an unpaired Student’s *t* test was used, while for comparisons among multiple groups, a one-way ANOVA was used. Data from TRAP-stained osteoclasts, the proportion of EdU-positive cells, and Western blot analyses were processed and quantified using ImageJ (version 1.54 g). Flow cytometry data were analyzed using FlowJo (version 10.8.1). Calcium imaging data were normalized and statistically analyzed using MATLAB (Version R2023b), and FRAP data were normalized using the FRAP Profiler V2 plugin in ImageJ. Statistical significance was set at *P* < 0.05. All data are expressed as means ± SD.

## Supplementary Materials

### This PDF file includes:

Supplementary Materials and Methods Figs. S1 to S50 Table S1 References
