# Supplementary material for: Oxidation-activated nanotherapy boosts tumor immunity and disrupts tumor-nerve crosstalk to combat bone metastases and cancer pain
Source: Sci Adv. 2026 Jan 23;12(4):eady1292. doi: 10.1126/sciadv.ady1292 (PMC12829578; doi:10.1126/sciadv.ady1292)
Supplement: Supplementary file 1 — Supplementary Materials and Methods Figs. S1 to S50 Table S1 References [file sciadv.ady1292_sm.pdf]

Science Advances
AAAS

Supplementary Materials for

Oxidation-activated nanotherapy boosts tumor immunity and disrupts tumor-nerve crosstalk to combat bone metastases and cancer pain

Zhaowei Zhang *et al.*

Corresponding author: Jiajia Xiang, xiang_jj@zju.edu.cn; Xin Liu, xinliuzju@zju.edu.cn; Shunwu Fan, shunwu_fan@zju.edu.cn; Youqing Shen, shenyq@zju.edu.cn

Sci. Adv. **12**, eady1292 (2026)
DOI: 10.1126/sciadv.ady1292

This PDF file includes:

Supplementary Materials and Methods
Figs. S1 to S50
Table S1
References

1. Materials and Methods

1.1 Materials

Acryloyl chloride, 2-(azepan-1-yl)ethan-1-ol, azodiisobutyronitrile, 4-cyano-4-(phenylcarbonothioylthio)pentanoic acid, and 4-(bromomethyl)phenylboronic acid were purchased from Bidepharm (Shanghai, China). All other reagents were purchased from Sinopharm Chemical Reagent Co. (Shanghai, China). Triethylamine (Et_3N), dichloromethane (DCM), dioxane, and dimethylformamide (DMF) were distilled over calcium hydride or dried using 4 Å molecular sieves before use. Branched poly(ethylenimine) (PEI, 25 kDa) was obtained from Sigma-Aldrich (Shanghai, China). Cell Counting Kit-8 (CCK-8) was sourced from APExBio (Houston, USA). The Bicinchoninic Acid (BCA) Protein Assay Kit, EdU Cell Proliferation Kit, ATP Assay Kit, Lactate Dehydrogenase (LDH) Assay Kit, Annexin V-FITC Apoptosis Detection Kit, Calcium Colorimetric Assay Kit, Fluo-4 Calcium Assay Kit, and Dihydroethidium (DHE) were purchased from Beyotime Biotechnology (Shanghai, China). The pVector plasmid (pHAGE) was purchased from MiaoLingBio (Wuhan, China). The pGSDMB plasmid (pHAGE-GSDMB) was generated as previously described (*74*). The Luciferase plasmid (pLuc) and Luciferase Assay System were acquired from Promega Corporation (Madison, USA). Mouse IFN-β ELISA Kits were obtained from Elabscience Biotechnology (Wuhan, China). The Ultrapure RNA Kit was sourced from CWBIO (Suzhou, China). Granzyme A (GZMA) protein was supplied by Sino Biological Inc. (Beijing, China). *Label* IT™ Cy5 Labelling Kit was procured from Mirus Bio LLC (Madison, USA). Transwell Chambers were obtained from Labselect (Beijing, China). The two-step immunohistochemistry detection kit and DAB Substrate Kit were purchased from Boster Biological Technology (Wuhan, China). 4T1 (mouse mammary carcinoma) and LLC (mouse Lewis lung carcinoma) cell lines were obtained from the Cell Bank of Type Culture Collection of the Chinese Academy of Science (Shanghai, China). The 4T1-Luc cell line was purchased from Sangon Biotech (Shanghai, China). The 4T1-GFP cell line was generated by stable transfection of 4T1 cells.

1.2 Synthesis of OPSA

2-(Azepan-1-yl)ethan-1-ol (2.00 g, 13.96 mmol) and Et_3N (2.12 g, 20.94 mmol) were dissolved in anhydrous DCM (50 mL) in a reaction flask maintained in an ice bath. Acryloyl chloride (1.52 g, 16.75 mmol) dissolved in DCM (20 mL) was added dropwise to the reaction mixture under constant stirring. After the addition, the reaction mixture was gradually warmed to room temperature and stirred overnight to ensure complete reaction. The reaction mixture was washed sequentially with water, saturated $NaHCO_3$ solution, and brine. The organic layer was separated and dried over anhydrous Na_2SO_4. The solvent was removed under reduced pressure using a rotary evaporator. The crude product was purified by vacuum distillation to yield 2-(azepan-1-yl)ethyl acrylate as a colorless oil (yield: 76%).

In an ampoule tube, 4-cyano-4-(phenylcarbonothioylthio)pentanoic acid (9.45 mg, 0.034 mmol), 2-(azepan-1-yl)ethyl acrylate (0.47 g, 2.37 mmol), and azodiisobutyronitrile (AIBN, 1.67 mg,

0.010 mmol) were dissolved in dioxane (2 mL). The mixture was degassed using three freeze-pump-thaw cycles and sealed under an argon atmosphere. The sealed tube was immersed in an oil bath preheated to 75 °C and stirred for 12 hours to complete the polymerization. The reaction was quenched by immersing the ampoule tube in liquid nitrogen. The product was precipitated three times in hexane, centrifuged, and dried under vacuum to yield poly(2-(azepan-1-yl)ethyl acrylate) (PSA) as a light yellow wax (yield: 70%).

PSA (0.2 g, 1.01 mmol tertiary amines) was dissolved in DMF (10 mL), and 4-(bromomethyl)phenylboronic acid (0.44 g, 2.03 mmol) was added to the solution. The reaction mixture was stirred at room temperature for 24 hours. The resulting solution was poured into tetrahydrofuran (THF) to precipitate the product. The precipitate was collected, washed with THF, and dried under a vacuum. The crude product was dissolved in water and dialyzed overnight against deionized water using a dialysis membrane with a molecular weight cutoff (MWCO) of 3,500 Da. The purified product was lyophilized to obtain OPSA as a white solid (yield: 68%).

1.3 Preparation and characterization of nanocomplexes (NCs)

OPSA was dissolved in HEPES buffer solution (10 mM, pH=7.4) at various concentrations. The solutions were added with the DNA solution of the same volume and vortexed for 10 seconds to produce the NCs at the desired N/P ratios. The polyplex solutions were incubated at room temperature for 30 minutes before characterization.

DNA binding ability was evaluated using an agarose gel retardation assay. The NCs were electrophoresed on a 1% agarose gel at 140 V for 30 minutes. DNA bands were visualized by staining with ethidium bromide excited by UV transillumination. The size and zeta potential of the polyplexes were measured at 25 °C using a Zetasizer Nano ZS (Malvern Instruments, UK). Data are presented as the means ± SD (n = 3). The morphology of polyplexes stained with phosphotungstic acid was visualized using a transmission electron microscope (TEM) (JEM-1200EX).

1.4 ROS responsiveness of OPSA and its NCs

The ROS responsiveness of OPSA and its nanocomplexes (NCs) was evaluated using a series of analytical techniques. For HPLC analysis, OPSA was dissolved in 100 μM H_2O_2 at 0.3 mg/mL and incubated at 37 °C. At predetermined time intervals, aliquots of 400 μL were sampled and analyzed using an Agilent HPLC system equipped with a 1260 Quat Pump VL, a 1260 VWD multi-λ fluorescence detector, a 1260 Vialsampler, and an Eclipse XDB-C18 column (4.6×250 mm, 5 μm). The eluent consisted of 10% methanol in water at a flow rate of 1.0 mL/minute. The release of p-hydroxymethylenephenol (HMP) was monitored, with pure HMP used as a standard, eluting at 7 minutes.

To confirm the chemical changes, OPSA (5 mg) was dissolved in 500 μL of CD_3OD and incubated with 10 mM H_2O_2 at 37 °C. Additional H_2O_2 was periodically introduced to compensate for its consumption, thereby maintaining a constant oxidative environment. Samples were taken at

timed intervals and analyzed using ^{1}H-NMR spectroscopy to characterize the structural transformation of OPSA.

The charge changes of OPSA/DNA NCs were monitored using dynamic light scattering (DLS). NCs prepared at an N/P ratio of 15 were incubated in HEPES buffer containing 100 μM H_2O_2 at pH 7.4 with gentle shaking. Samples were taken at specific time points, and their zeta potentials were measured.

To evaluate DNA release under oxidative conditions, OPSA/DNA NCs prepared at an N/P ratio of 15 were incubated with varying H_2O_2 concentrations (0, 5, 10, 20, 40, 100, and 200 μM) for 1 hour at 37 °C. Naked DNA and DNA incubated with 100 μM H_2O_2 were used as controls. The samples were subjected to gel electrophoresis, as previously described, to assess DNA release from the NCs. The size of the NCs after incubation with 100 μM H_2O_2 for 2 hours was measured using DLS on a Zetasizer Nano ZS.

1.5 Preparation and characterization of fusogenic liposomal NCs (LipoNCs)

Fusogenic liposomes were prepared using the thin-film hydration method, following established protocols (*37*). The molar ratio of DOPE/CHEMS/DSPE-PEG2000 was fixed at 7.65:2:1.35. DOPE (11.06 mg), CHEMS (1.88 mg), and DSPE-PEG2000 (7.35 mg) were dissolved in 2 mL chloroform, and the organic solvent was evaporated under reduced pressure to form a thin lipid film. The lipid film was hydrated overnight with 1 mL HEPES buffer (10 mM, pH 7.4) at room temperature with gentle stirring. To achieve a uniform suspension, the hydrated liposome solution was sonicated in an ice bath for 10 minutes. The resulting liposome solution was filtered through a 0.22 μm membrane to remove aggregates. For the preparation of LipoNCs, 200 μL of the liposome solution was added to a suspension of NCs (N/P ratio of 15, containing 200 μL of NC solution and 4 μg of DNA). The mixture was incubated overnight at room temperature to allow the liposomes to coat the NCs, forming fusogenic LipoNCs.

The particle size and zeta potential of the LipoNCs were measured using DLS. The morphology of the LipoNCs was visualized using cryogenic TEM (cryo-TEM) with a Talos F200C instrument.

1.6 *In vitro* gene transfection

The Luciferase reporter assay was first employed to evaluate the transfection efficiency of NCs. 4T1 cells were seeded in 96-well plates at a density of 1.5×10^4 cells per well and incubated for 24 hours. Once adhered, the complete medium was replaced with serum-free DMEM, and NCs containing 0.5 μg of pLuc were added for transfection. After 4 hours, the medium was replaced with complete DMEM, and the cells were incubated for an additional 48 hours. Cells were then lysed, and luciferase expression was quantified using a luciferase assay kit. For oxidative stress conditions, the same procedure was followed, except the pLuc content in the transfection mixture was reduced to 0.1 μg. Following the 4-hour transfection period, the medium was replaced with complete DMEM containing H_2O_2 at 0, 10, 50, 100, or 200 μM. For pGSDMB transfection, 4T1 cells were seeded in 6-well plates at a density of 3×10^5 cells per well and incubated for 24 hours. The complete medium was then replaced with serum-free DMEM, and NCs or LipoNCs

containing 4 μg of pGSDMB were added for a 4-hour incubation. The medium was subsequently replaced with complete DMEM, with or without H_2O_2, and the cells were incubated for 48 hours. Cell lysates were collected for Western blot analysis.

1.7 Subcellular distribution of NCs in 4T1 cells

4T1 cells were seeded at a density of 1×10^4 cells per well in confocal dishes and incubated at 37 °C for 24 hours to allow adhesion. OPSA/^{Cy5}pLuc NCs containing 0.5 μg of DNA were added to each well and incubated for 4 hours. Lysosomes were stained with LysoTracker Green (1: 1000) for 15 minutes, and nuclei were counterstained with Hoechst 33342 (1: 1000) for 10 minutes. After staining, cells were washed twice with phosphate-buffered saline (PBS), and fluorescence images were captured using confocal laser scanning microscopy (CLSM, Zeiss LSM 880). The fluorescence excitation/emission settings used were Cy5 (Ex. 633 nm, Em. 667 nm), LysoTracker Green (Ex. 488 nm, Em. 523 nm), and Hoechst 33342 (Ex. 405 nm, Em. 458 nm).

1.8 Membrane fusion assessment

To evaluate membrane fusion-mediated internalization of LipoNCs, 4T1 cells were seeded in confocal dishes at a density of at a density of 1×10^4 cells per well and incubated at 37 °C for 24 hours to allow adhesion. Cells were then treated with dual-labeled LipoNCs, consisting of DiI-labeled liposomes and Cy5-labeled plasmid DNA (^{Cy5}DNA), and incubated for either 1 hour or 8 hours. For the 1-hour group, cells were washed with PBS and immediately imaged. For the 8-hour group, cells were washed after 1 hour of incubation and then further cultured in fresh complete medium for an additional 7 hours before imaging. To visualize subcellular distribution, lysosomes were stained with LysoTracker Green (1:1000 dilution) for 15 minutes, followed by nuclear staining with Hoechst 33342 (1:1000 dilution) for 10 minutes. After washing, cells were imaged using CLSM. Excitation/emission settings were as follows: Cy5 (Ex: 633 nm, Em: 667 nm), DiI (Ex: 549 nm, Em: 565 nm), LysoTracker Green (Ex: 488 nm, Em: 523 nm), and Hoechst 33342 (Ex: 405 nm, Em: 458 nm).

1.9 Uptake pathway analysis

To determine the cellular internalization pathways of LipoNCs, pharmacological inhibition assays were performed. 4T1 cells were seeded in 6-well plates at a density of 2×10^5 cells per well and cultured for 24 hours. Subsequently, filipin (7.5 μM), wortmannin (5 μM), chlorpromazine (50 μM), or cytochalasin D (5 μM) were added to the culture medium, and the cells were incubated at 37°C for 30 minutes. After pretreatment, the inhibitor-containing medium was removed, and cells were washed and incubated with fresh complete medium containing LipoNCs loaded with Cy5-labeled OPSA (^{Cy5}OPSA) for 4 hours. For the low-temperature control group, cells were incubated with LipoNCs at 4 °C. After incubation, all cells were washed with cold PBS, detached by trypsinization, resuspended in PBS, and analyzed by flow cytometry

1.10 *In vivo* cellular uptake assay of LipoNCs.

To assess the cellular uptake of LipoNCs within tumor tissues, an intra-femoral bone metastasis model was established by injecting 4T1-GFP cells into the femoral bone marrow cavity of BALB/c mice. Two weeks post-inoculation, mice were intravenously administered ^{Cy5}OPSA-formulated LipoNCs at a DNA dose of 1 mg/kg. At 24 hours post-injection, tumors were excised, finely minced into ~1 mm × 1 mm pieces, and enzymatically digested in a solution containing collagenase type IV (200 U/mL) and DNase I (100 μg/mL) at 37 °C for 1 hour with gentle agitation. The digestion was terminated by adding fetal bovine serum (FBS), and the resulting mixture was passed through a 40 μm mesh to obtain a single-cell suspension. The cell suspensions were analyzed by flow cytometry to determine the cellular identity of LipoNCs-positive cells within the tumor microenvironment.

1.11 CCK-8 cytotoxicity assay

4T1 cells were seeded in 96-well plates at a density of 3×10^3 per well and incubated for 24 hours. OPSA NCs containing 0.5 μg of pLuc at N/P ratios of 5, 10, 15, or 20 were added to the wells in a serum-free medium. After a 4-hour incubation, the medium was replaced with complete DMEM, and the cells were cultured for an additional 48 hours. The medium was then replaced with fresh complete DMEM containing 10% CCK-8 solution in each well. A cell-free control group was included. After incubation at 37 °C for 2 hours, the absorbance at 450 nm was measured using a microplate reader to assess cell viability.

1.12 Western blot analysis

For cell samples, cells were scraped with a cell scraper and lysed in RIPA lysis buffer supplemented with protease and phosphatase inhibitors, centrifuged at 12,000 rpm for 15 minutes at 4 °C. Tumor tissue samples were homogenized in RIPA lysis buffer and filtered through a 40 μm mesh to obtain a uniform protein solution. The protein concentration of the samples was quantified using the BCA Protein Assay Kit. Denaturing loading buffer (5×) was added to the lysed protein samples, which were then denatured at 95 °C for 10 minutes. Next, 20 μg of denatured protein were separated by 10% sodium dodecyl sulfate-polyacrylamide gel electrophoresis (SDS-PAGE) and transferred to polyvinylidene difluoride (PVDF) membranes. The membranes were blocked with 5% BSA in Tris-buffered saline and incubated sequentially with primary and secondary antibodies. Protein bands were detected using an enhanced chemiluminescent (ECL) substrate (Fude Biological Technology Co., Ltd., cat. no. FD8020, China) and imaged under FUSION FX Bio Imaging System (VILBER, Paris, France).

1.13 Intracellular calcium levels measurement

4T1 cells were seeded in 6-well plates at a density of 1.5×10^5 per well and incubated for 24 hours. ^{PEI}LipoNCs or ^{OPSA}LipoNCs containing 4 μg of pVector were added to the wells in a serum-free medium. After a 4-hour incubation, the medium was replaced with complete DMEM, and the cells were cultured for an additional 48 hours. Afterward, 4T1 cells were collected and lysed, and their

calcium content was measured using the Calcium Colorimetric Assay Kit (Beyotime, China). The calcium ion content was normalized to the control group for statistical analysis.

1.14 RNA extraction and quantitative real-time PCR

Total RNA was extracted from cells, bone marrow, and tumor tissues using the Ultrapure RNA Kit (Cwbio) following the manufacturer's instructions. The RNA was then reverse-transcribed into complementary DNA (cDNA) using the PrimeScript™ RT Reagent Kit (Accurate Biotechnology). Quantitative PCR (qPCR) was performed with Hieff® qPCR SYBR Green Master Mix (Yeason). Mouse-specific primers used in the qPCR are listed in Supplementary Table 1. GAPDH was used as the internal control for statistical analysis.

1.15 RNA sequencing

For RNA sequencing (RNA-seq), 4T1-GFP cells were used to establish a mouse bone metastasis model. At the end of the treatment period, the mice were euthanized by carbon dioxide asphyxiation. Tumors were isolated, minced into 1 mm × 1 mm fragments, and digested with collagenase type IV (200 U/mL) and DNase I (100 µg/mL) at 37 °C for 1 hour. The digestion was terminated by adding FBS, and the remaining tissue was filtered through a 40 µm mesh. The tumor cell suspension was then sorted using flow cytometry on a CytoFLEX SRT flow cytometer (Beckman Coulter, Brea, CA, USA), and GFP-positive cells were isolated after debris removal. The cells were centrifuged and lysed using a TRIzol reagent.

RNA extraction, sequencing, and statistical analysis were performed by Shanghai Majorbio Bio-pharm Biotechnology Co., Ltd. (Shanghai, China). The RNA-seq transcriptome library was prepared using the Illumina® Stranded RNA Prep Ligation protocol (Illumina, San Diego, CA). Transcript expression levels were quantified using the transcripts per million reads (TPM) method. Genes with an adjusted P-value (<0.05) and an absolute log2 fold change (≥ 1) were considered differentially expressed. GO functional enrichment and KEGG pathway analysis were conducted using Goatools and KOBAS (*75*), respectively.

1.16 Tumor spheroid formation

4T1 tumor spheroids were generated using the hanging-drop method (*76*). Briefly, 4T1 cells were diluted in 10% FBS DMEM to a concentration of 5×10^4 cells/mL. Twenty-microlitre droplets, each containing approximately 1×10^3 cells, were placed on the inner lid of a culture dish, enabling cell aggregation and spheroid formation under gravity. After 48 hours of incubation, stable spheroids were collected and individually transferred to agarose-coated 96-well plates.

1.17 Immunophenotyping analysis

Spleens, TDLNs, and tumor tissues were collected from mice at the experimental endpoint for immunophenotyping. For the spleens and TDLNs, single-cell suspensions were prepared by grinding spleens and TDLNs through a 40-µm cell strainer, while tumor tissues were

enzymatically digested with collagenase IV and DNase I to generate suspensions. The cells were washed with PBS containing 0.5% BSA and 5 mM EDTA (PBS/BSA/EDTA) and fixed with 4% PFA for 10 minutes at room temperature. After fixation, cells were washed with PBS/BSA/EDTA and resuspended for antibody staining. Flow cytometry analysis included identification of $CD4^+$ T cells ($CD3^+CD4^+$), $CD8^+$ T cells ($CD3^+CD8^+$), central memory T cells ($CD44^+CD62L^+$), effector memory T cells ($CD44^+CD62L^-$), mature DCs ($CD11c^+CD80^+CD86^+$), Tregs ($CD3^+CD4^+FOXP3^+$), and activated CTLs ($CD3^+CD8^+IFN\text{-}\gamma^+$). All samples were analyzed using a CytoFLEX S flow cytometer (Beckman Coulter, Brea, CA, USA), and data were processed with FlowJo software (FlowJo). Detailed information on antibodies, their working concentrations, and the gating strategy is provided in the Supplementary Information.

1.18 Immunofluorescence and immunohistochemistry assay

Tumor samples and tumor-bearing bone tissues were fixed in 4% PFA for 24 hours at room temperature. The bone tissues were then decalcified in 10% EDTA solution at room temperature for 7 days, with daily solution replacement. Both tissue types were subsequently washed with PBS, paraffin-embedded, and sectioned into 5-μm-thick slices for staining.

Immunofluorescence staining: Paraffin sections underwent antigen retrieval with 0.1% trypsin at 37 °C for 1 hour. Endogenous peroxidase activity was blocked by incubating sections with hydrogen peroxide (Beyotime) for 10 minutes. Nonspecific binding was blocked with a Beyotime blocking solution at room temperature for 1 hour. Sections were then incubated overnight at 4 °C with primary antibodies. Then, sections were incubated with fluorescently conjugated secondary antibodies for 1 hour at room temperature. After washing with Tris-Buffered Saline with Tween-20 (TBST), sections were mounted with an anti-fade medium and observed under a fluorescence microscope.

Immunohistochemistry staining: The procedures for antigen retrieval, endogenous peroxidase quenching, and blocking were identical to those used in immunofluorescence. Sections were incubated overnight at 4 °C with primary antibodies, followed by incubation at 37 °C for 30 minutes with HRP-conjugated secondary antibodies. After TBST washes, sections were incubated with Diaminobenzidine (DAB) substrate at room temperature for 5 minutes to visualize the antigen-antibody complex. Finally, sections were counterstained with hematoxylin, mounted, and examined under a light microscope.

TRAP staining: Bone tissue sections were stained with TRAP solution (HaoKe Biotechnology) at room temperature for 2 hours, then washed with TBST and mounted. Red to orange-stained osteoclasts were observed under a light microscope.

1.19 Antibody usage

The following antibodies were used for flow cytometry: PE anti-mouse CD3 (BioLegend, Cat. No. 100205, 1:200), PerCP-Cy5.5 anti-mouse CD4 (BioLegend, Cat. No. 100433, 1:200), FITC anti-mouse CD8 (BioLegend, Cat. No. 100803, 1:200), FITC anti-mouse CD80 (BioLegend, Cat. No. 104705, 1:200), APC anti-mouse CD86 (BioLegend, Cat. No. 159215, 1:200), PE anti-mouse

CD11c (BioLegend, Cat. No. 117307, 1:200), PE-Cy7 anti-mouse CD44 (BioLegend, Cat. No. 103029, 1:200), and FITC anti-mouse CD62L antibodies (BioLegend, Cat. No. 161211, 1:200), APC anti-mouse Foxp3 (Elabscience, E-AB-F1238E, 1:200) and PE/Elab Fluor® 594 anti-mouse IFN-γ antibodies (Elabscience, E-AB-F1101P, 1:200).

The following primary antibodies were used for Western blot analysis: anti-STING (Affinity, DF12090, 1:1000), anti-p-STING (Affinity, AF7416, 1:1000), anti-TBK1 (Affinity, DF7026, 1:1000), anti-p-TBK1 (Affinity, AF8190, 1:1000), anti-IRF3 (Affinity, DF6895, 1:1000), anti-p-IRF3 (Affinity, AF2436, 1:1000), anti-GSDMB (Abcam, ab215729, 1:1000), anti-CACNA1A (Abcam, ab32642, 1:1000), anti-CACNA1C (Abcam, ab84814, 1:1000), and anti-CACNA1G (Affinity, DF10014, 1:1000).

For immunofluorescence staining, the following antibodies were employed: anti-p-STING (Affinity, AF7416, 1:200), anti-CD8 (Abcam, ab237709, 1:100), anti-GZMA (Proteintech, 11288-1-AP, 1:100), anti-Ki67 (Affinity, AF0198, 1:100), anti-CGRP (Abcam, ab283568, 1:200), anti-TRPV1 (Abcam, ab203103, 1:200), and anti-Substance P (Abcam, ab14184, 1:100).

The antibodies used for immunohistochemistry analysis were as follows: anti-CACNA1A (Proteintech, 27227-1-AP, 1:50), anti-CACNA1C (Proteintech, 21774-1-AP, 1:100), and anti-CACNA1G (Proteintech, 17821-1-AP, 1:50).

2. Supporting Figures and Tables

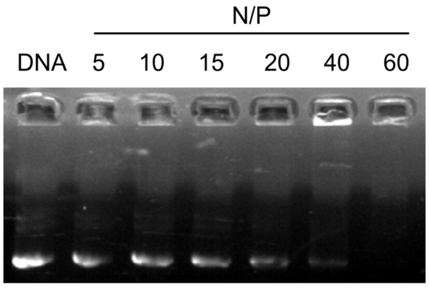

fig. S1. Gel retardation assay of PSA/DNA NCs at varying N/P ratios.

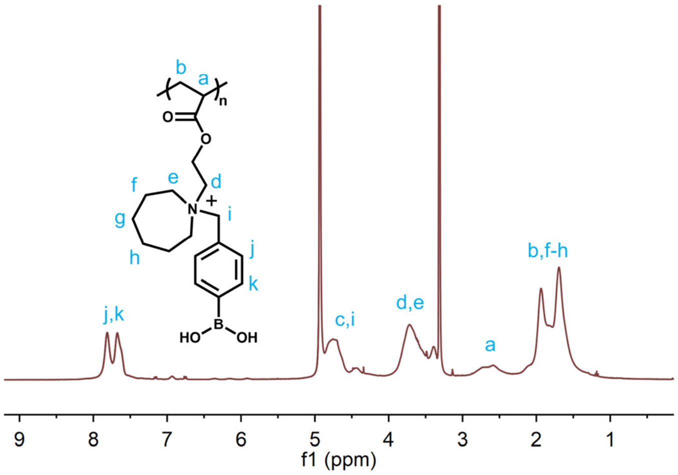

fig. S2. The ^{1}H-NMR spectra of OPSA in CD_3OD.

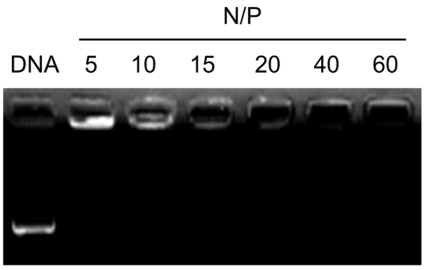

fig. S3. Gel retardation assay of OPSA/DNA NCs at varying N/P ratios.

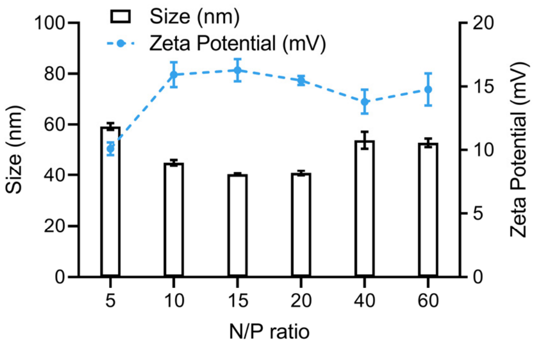

fig. S4. Size and zeta potential of NCs at different N/P ratios.

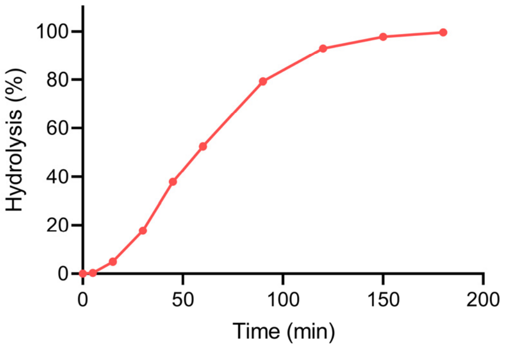

fig. S5. Hydrolysis kinetics of OPSA under oxidative conditions. OPSA (0.3 mg/mL) was incubated with 100 μM H_2O_2 at 37 °C, and the release of p-hydroxymethylenephenol (HMP) was monitored over time using HPLC.

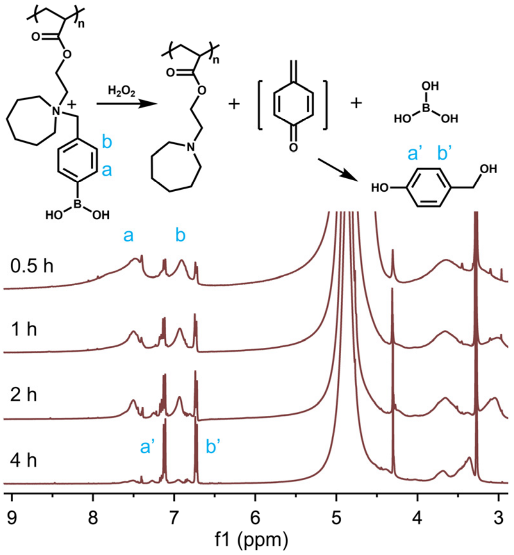

fig. S6. H_2O_2-responsive oxidation and hydrolysis of the phenylboronic acid group in OPSA detected by ^{1}H-NMR spectroscopy. OPSA was dissolved in CD_3OD and treated with 10 mM H_2O_2, with measurements taken at various time intervals.

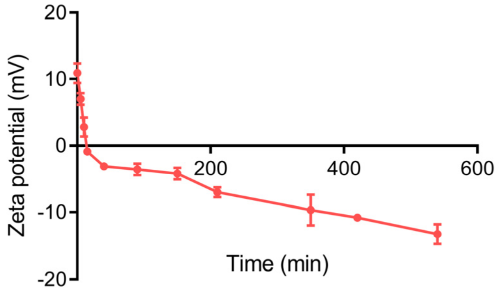

fig. S7. Zeta potential changes of OPSA NCs under oxidative hydrolysis. NCs prepared at an N/P ratio of 15 were incubated with 100 µM H_2O_2.

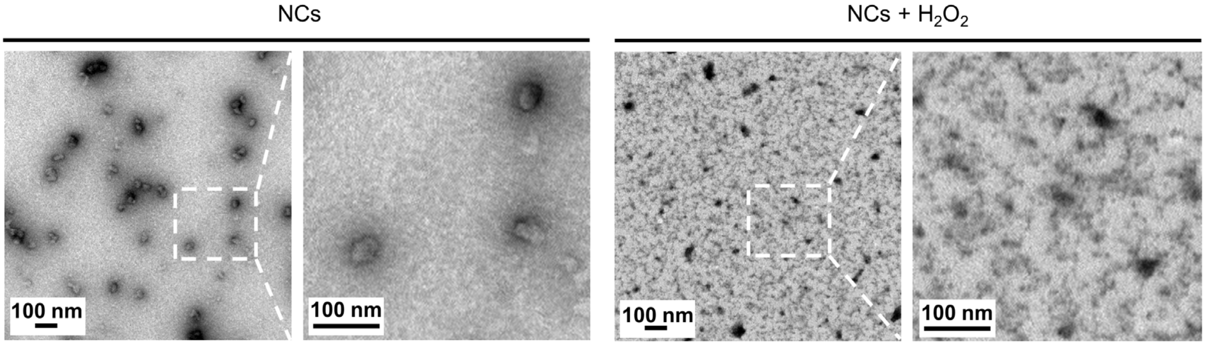

fig. S8. Transmission electron microscopy (TEM) results of OPSA NCs under normal or oxidative conditions. NCs were incubated with or without 100 µM H_2O_2 stimulation for 2 hours.

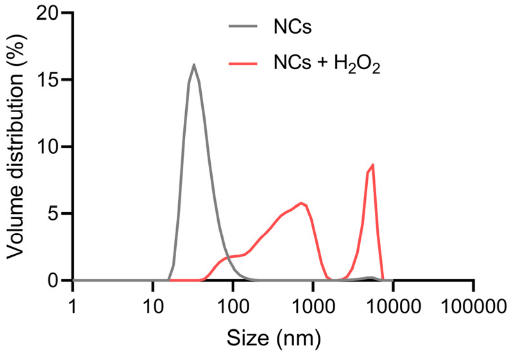

fig. S9. Size distribution of OPSA NCs under normal or oxidative conditions. NCs were incubated with or without 100 µM H_2O_2 stimulation for 2 hours.

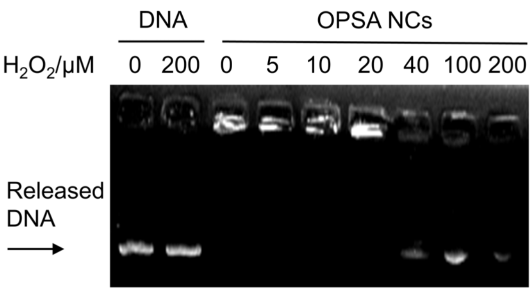

fig. S10. Gel retardation assay of OPSANCs under normal or oxidative conditions. NCs at an N/P ratio of 15 were incubated with varying concentrations of H_2O_2 for 1 hour.

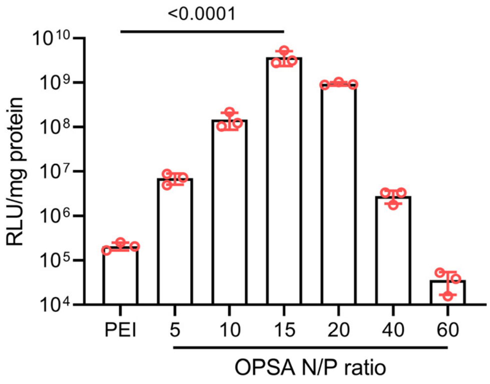

fig. S11. Transfection efficiency of pLuc-loaded OPSA NCs at varying N/P ratios. PEI (N/P = 7) was used as a positive control. Statistical significance was assessed using one-way ANOVA, and data are presented as mean ± SD; n = 3.

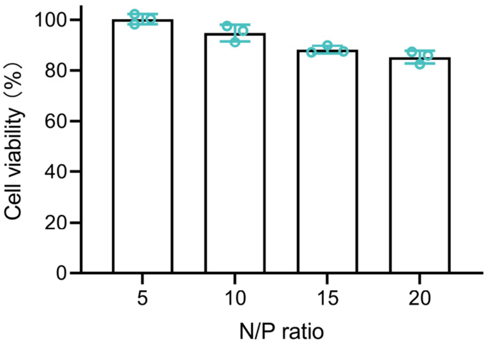

fig. S12. Cytotoxicity of OPSA NCs. Cell viability of 4T1 cells treated with OPSA NCs at varying N/P ratios was assessed by the CCK-8 assay after 48 hours of incubation. Data are presented as mean ± SD; n = 3.

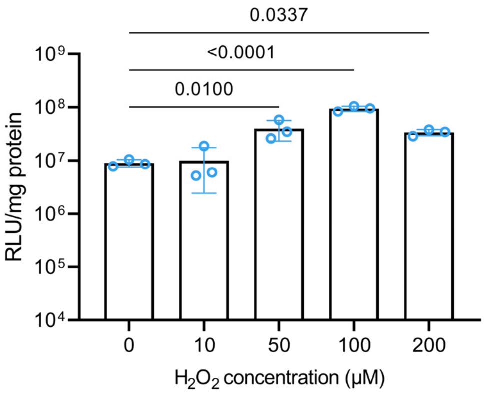

fig. S13. The transfection efficiency of pLuc-loaded OPSA NCs (N/P = 15) in 4T1 cells under varying H_2O_2 concentrations. Statistical significance was determined using one-way ANOVA. Data are presented as mean ± SD; n = 3.

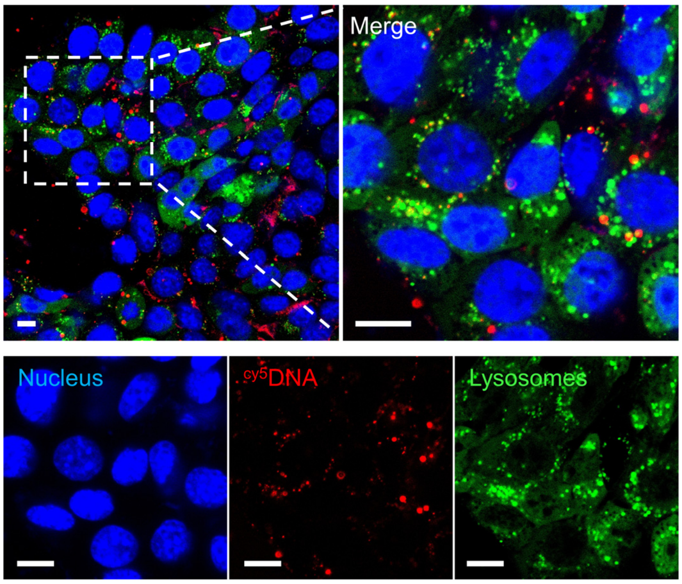

fig. S14. Subcellular distribution of OPSA NCs in 4T1 cells. 4T1 cells were incubated with ^{Cy5}DNA-loaded OPSA NCs in a serum-free medium for 4 hours and observed using confocal laser scanning microscopy (CLSM). Cy5-labeled DNA (^{Cy5}DNA) is shown in red, lysosomes stained with LysoTracker Green are visualized in green, and cell nuclei stained with Hoechst 33342 appear in blue. Scale bar, 10 μm.

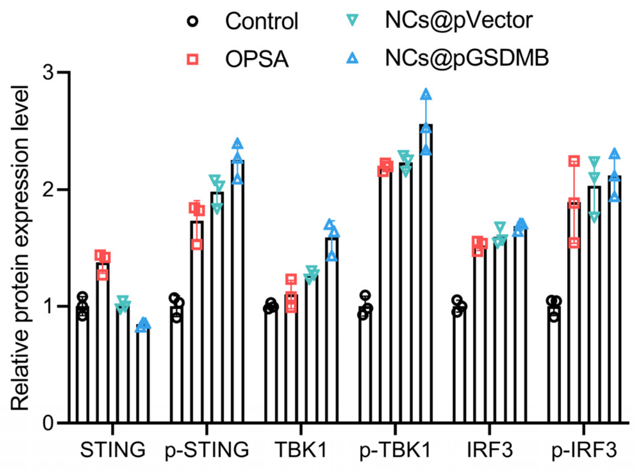

fig. S15. Assessment of STING pathway activation in 4T1 cells. Quantification of expression and phosphorylation levels of STING, TBK1, and IRF3 in 4T1 cells across different treatments.

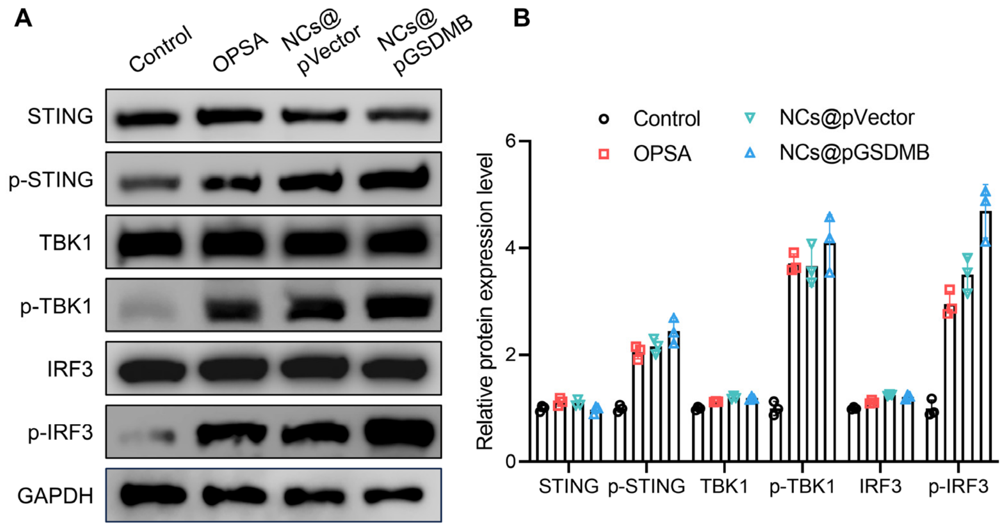

fig. S16. Assessment of STING pathway activation in LLC cells. Expression and phosphorylation levels of STING, TBK1, and IRF3 in LLC cells across different treatments (**A**) and quantification (**B**).

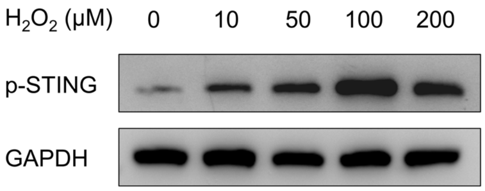

fig. S17. Assessment of STING activation of NCs upon H_2O_2 stimulation. The expression of phosphorylated STING (p-STING) in 4T1 cells induced by OPSA NCs under different concentrations of H_2O_2 was evaluated using Western blot analysis.

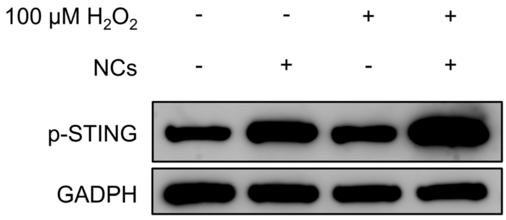

fig. S18. The effect of H_2O_2 on STING activation. The expression levels of p-STING in 4T1 cells were assessed by Western blot analysis following treatment with NCs, 100 μM H2O2, or both.

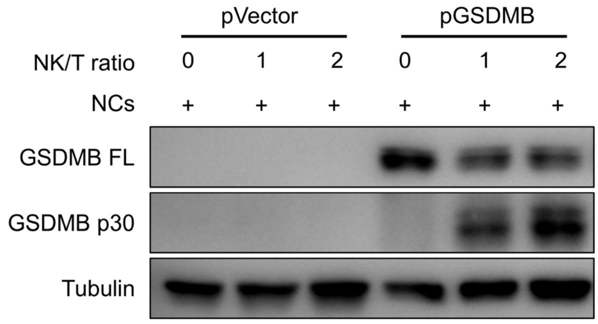

fig. S19. Assessment of immune cell-mediated pyroptotic events. Expression levels of full-length (FL) GSDMB and its active p30 fragment were evaluated using Western blot analysis in transfected 4T1 cells after co-culture with NK cells at varying NK-to-tumor cell (NK/T) ratios.

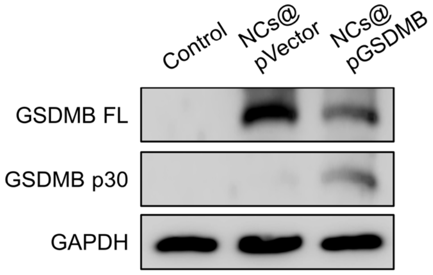

fig. S20. Assessment of GSDMB-mediated pyroptosis in LLC cells. Expression levels of GSDMB FL and its active p30 fragment in LLC cells were evaluated using Western blot analysis post-pyroptosis simulation *via* electroporation-mediated GZMA delivery.

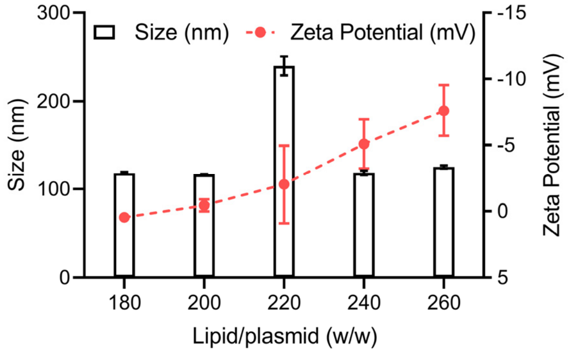

fig. S21. Particle size and zeta potential of LipoNCs at varying lipid-to-plasmid ratios. Data are presented as mean ± SD; n = 3.

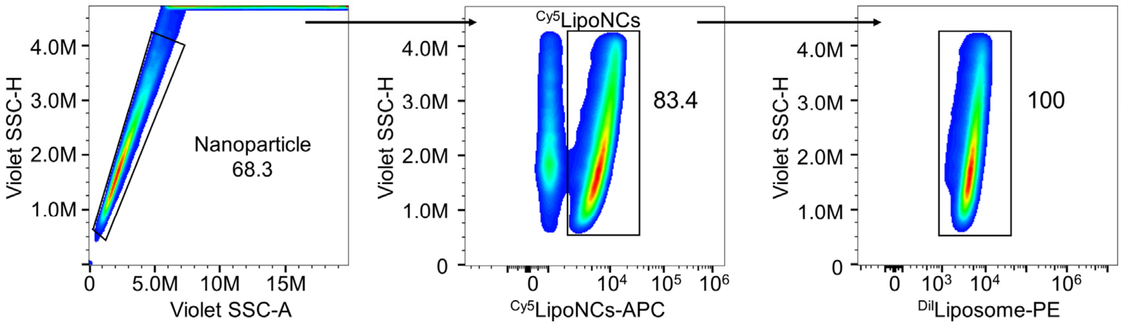

fig. S22. Nanoflow cytometry analysis of the encapsulation efficiency of LipoNCs. NCs were formed through complexation of Cy5-labeled OPSA (^{Cy5}OPSA) with DNA, and liposomes were labeled with DiI.

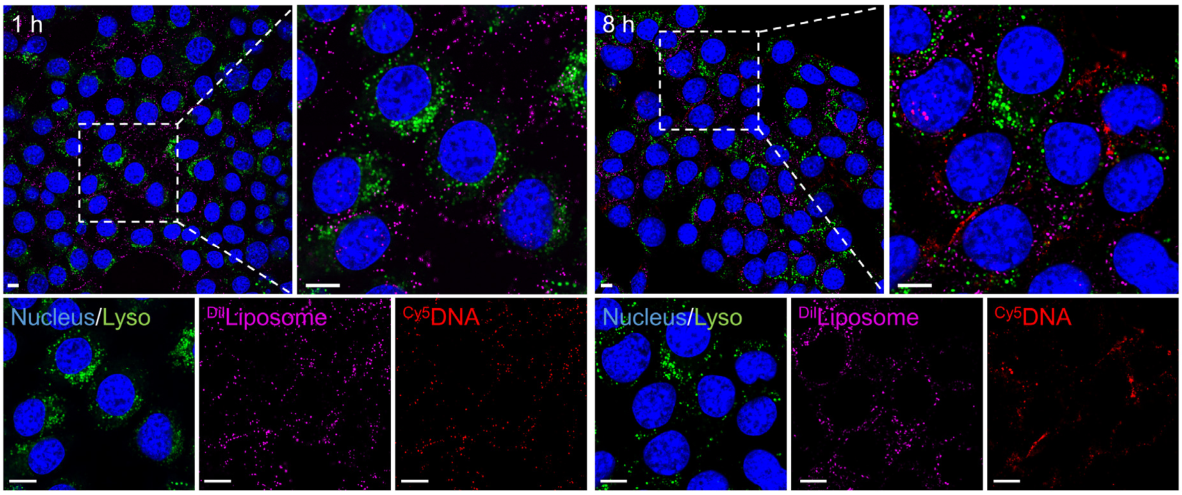

fig. S23. Confocal fluorescence images of membrane fusion-mediated uptake of LipoNCs by 4T1 cells at 1 hour and 8 hours. ^{Cy5}DNA is shown in red, lysosomes stained with LysoTracker Green are visualized in green, DiI-labeled liposome is shown in pink, and cell nuclei stained with Hoechst 33342 appear in blue. Scale bar, 10 µm.

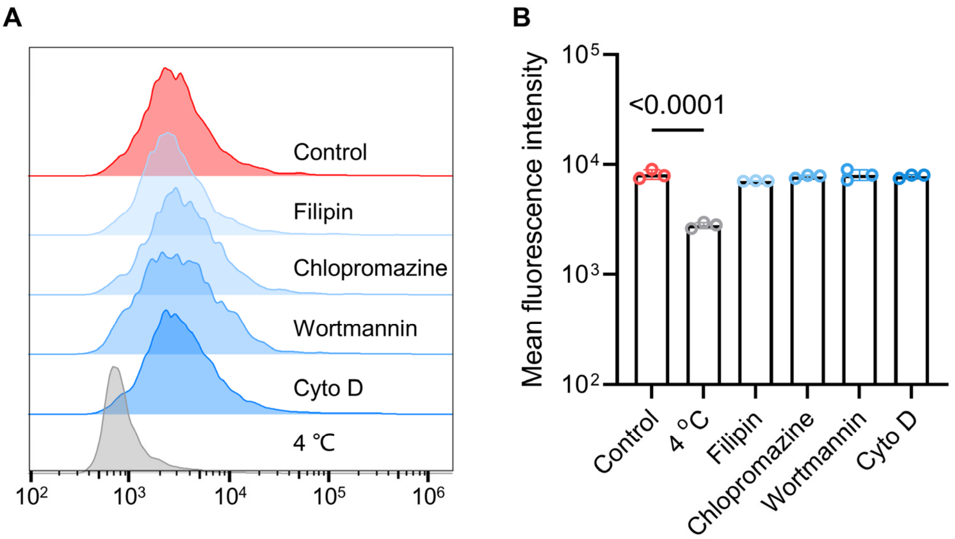

fig. S24. Internalization pathways of LipoNCs. (**A**) Flow cytometric analysis of LipoNCs uptake by 4T1 cells under different endocytic inhibitors or at 4 °C. (**B**) Quantification of Cy5 mean fluorescence intensity in each group.

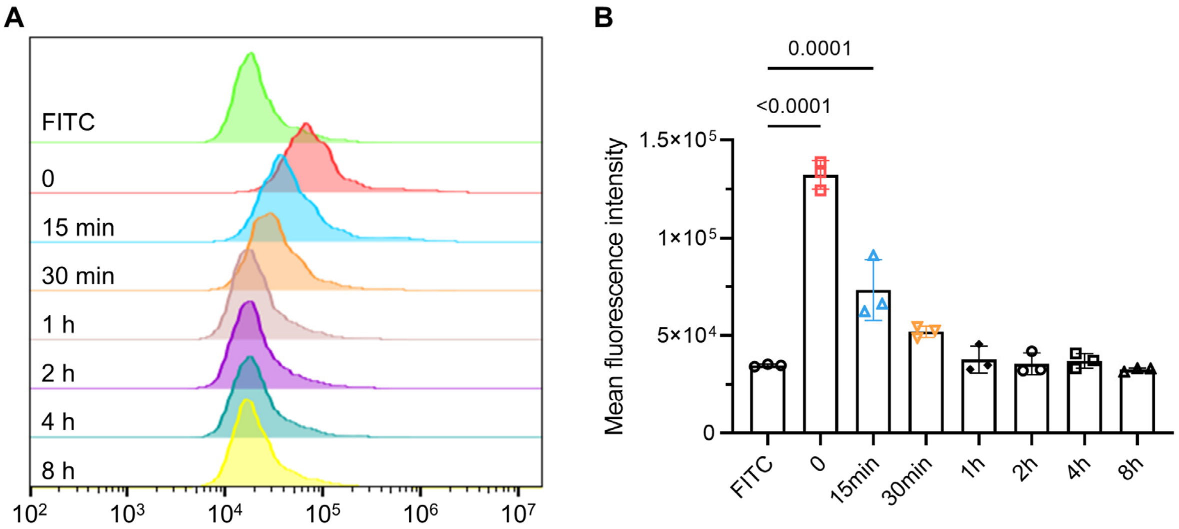

fig. S25. The membrane permeabilization to FITC dye over time after removal of LipoNCs. (**A**) Flow cytometry analysis of FITC fluorescence intensity in ^{OPSA}LipoNCs-treated 4T1 cells. At various time intervals post-removal of LipoNCs, cells were incubated with FITC dye for 1 hour. (**B**) Quantitative analysis of FITC fluorescence intensity in 4T1 cells. Data are presented as mean ± SD; n = 3. Statistical significance was assessed using one-way ANOVA.

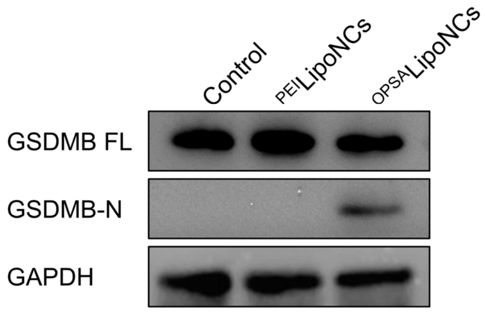

fig. S26. Assessment of pyroptotic events facilitated by ^{OPSA}LipoNCs. Western blotting analysis was performed to detect cleaved GSDMB in GSDMB-overexpressing 4T1 cells after GZMA delivery *via* ^{PEI}LipoNCs or ^{OPSA}LipoNCs.

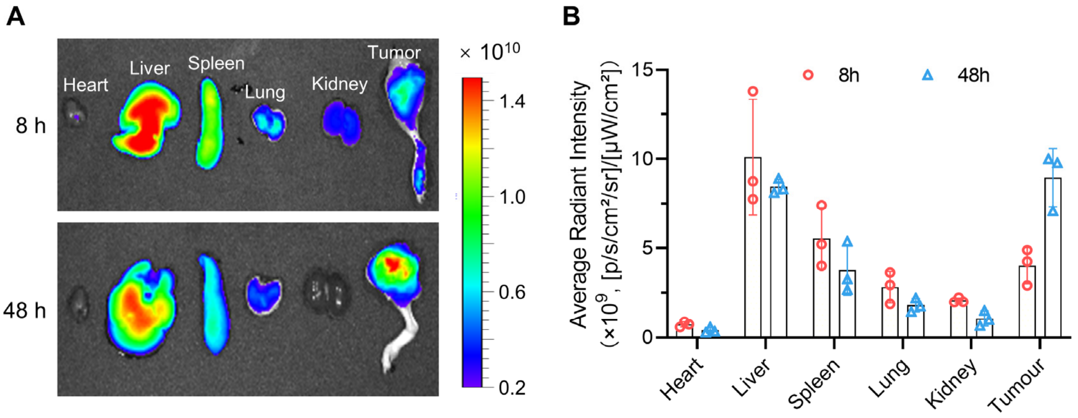

fig. S27. *In vivo* distribution of ^{OPSA}LipoNCs. (**A**) Representative *ex vivo* fluorescence images of major organs at different time points following intravenous administration of DiR-labelled ^{OPSA}LipoNCs. (**B**) Quantitative analysis of DiR fluorescence intensity in various organs over time. Data are presented as mean ± SD; n = 3.

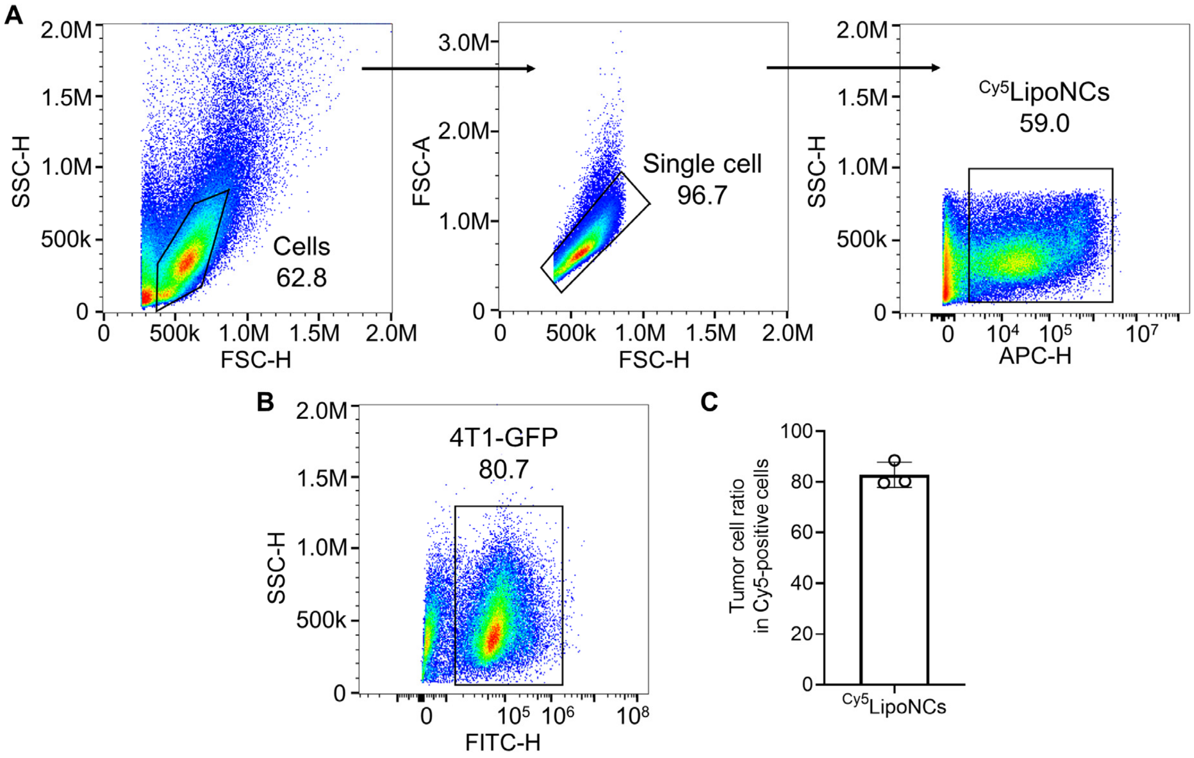

fig. S28. Assessment of cellular uptake of ^{Cy5}LipoNCs in tumor tissues. (**A**) Flow cytometry gating strategy for identifying cells that internalized Cy5-labeled LipoNCs (^{Cy5}LipoNCs). (**B, C**) Flow cytometry analysis (**B**) and quantification (**C**) of the percentage of 4T1-GFP cells among those internalized ^{Cy5}LipoNCs in tumors; n = 3.

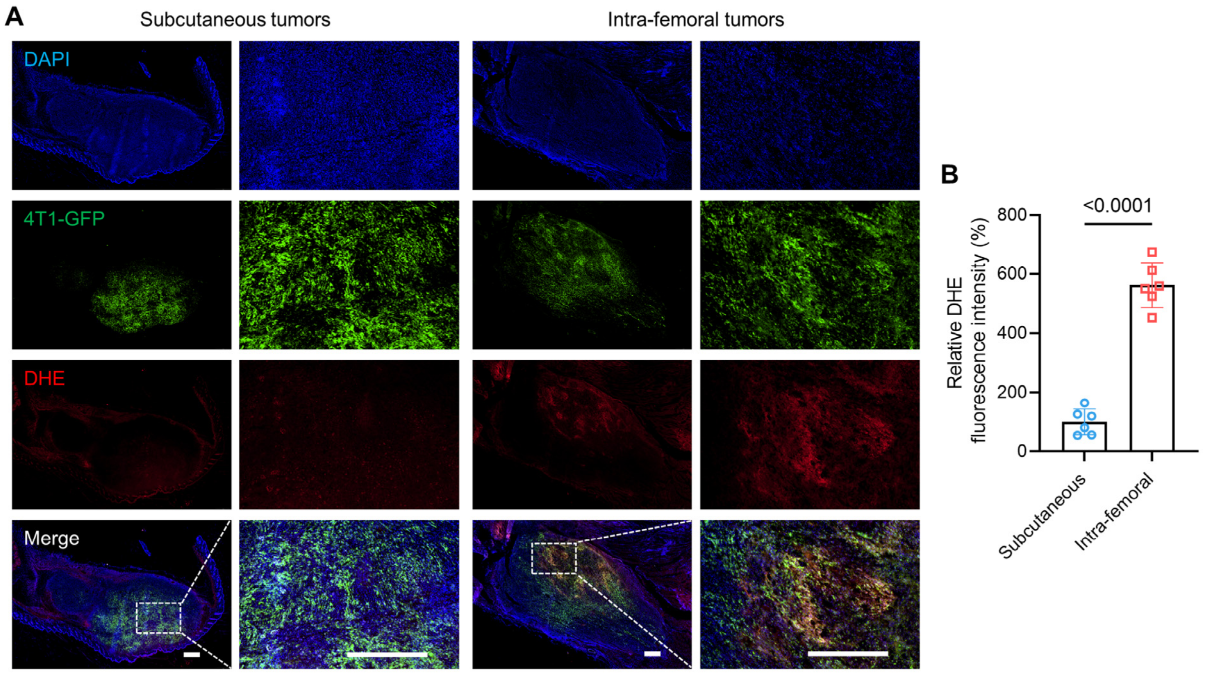

fig. S29. Comparison of ROS levels in subcutaneous and intra-femoral tumors. (**A**) Representative fluorescence images. (**B**) Quantification of relative fluorescence intensity of dihydroethidium (DHE) staining; n = 6. ROS indicated by DHE is shown in red, 4T1-GFP cells are shown in green, and nuclei stained with DAPI are shown in blue. Scale bar, 500 μm.

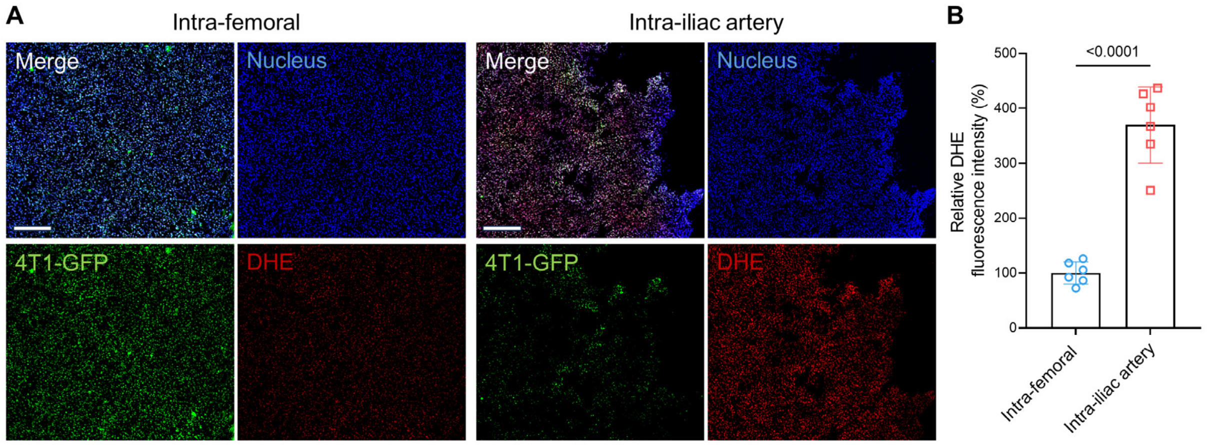

fig. S30. Comparison of ROS levels in bone metastases induced by intra-femoral and intra-iliac artery injections. (**A**) Representative fluorescence images. (**B**) Quantification of relative fluorescence intensity of DHE staining; n = 6. ROS indicated by DHE is shown in red, 4T1-GFP cells are shown in green, and nuclei stained with DAPI are shown in blue. Scale bar, 500 μm.

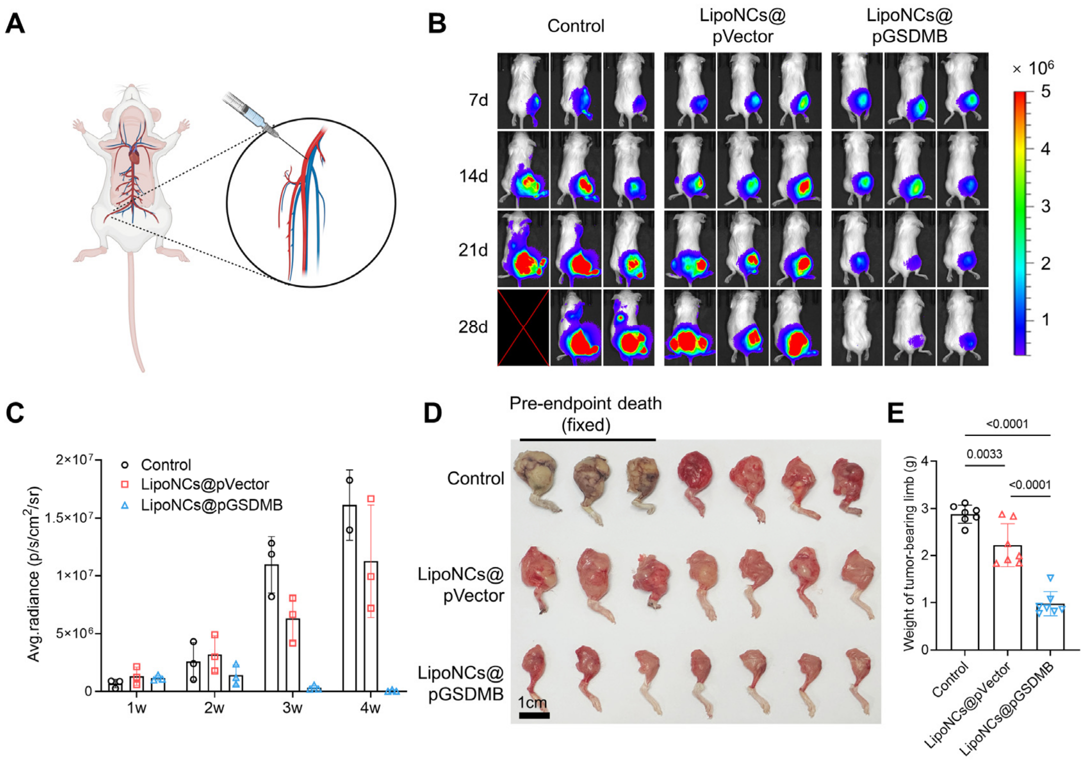

fig. S31. The antitumor efficacy of LipoNCs in the intra-iliac artery bone metastasis model. (**A**) Schematic illustration of iliac artery injection for bone metastasis modeling. 4T1-GFP tumor cells (1 × 10^5 cells) were injected into the iliac artery using a 31G insulin syringe. "Created in BioRender. Zhang, Z. (2026) https://BioRender.com/fz6yd7k "(**B**) *In vivo* bioluminescence imaging of bone metastases in anesthetized mice following treatment; n = 3. (**C**) Quantification of the averaged bioluminescence intensity over time for each treatment group. (**D**) Images of tumor-bearing limbs collected at the study endpoint; n = 7. (**E**) Weight of tumor-bearing limbs measured across different treatment groups; n = 7.

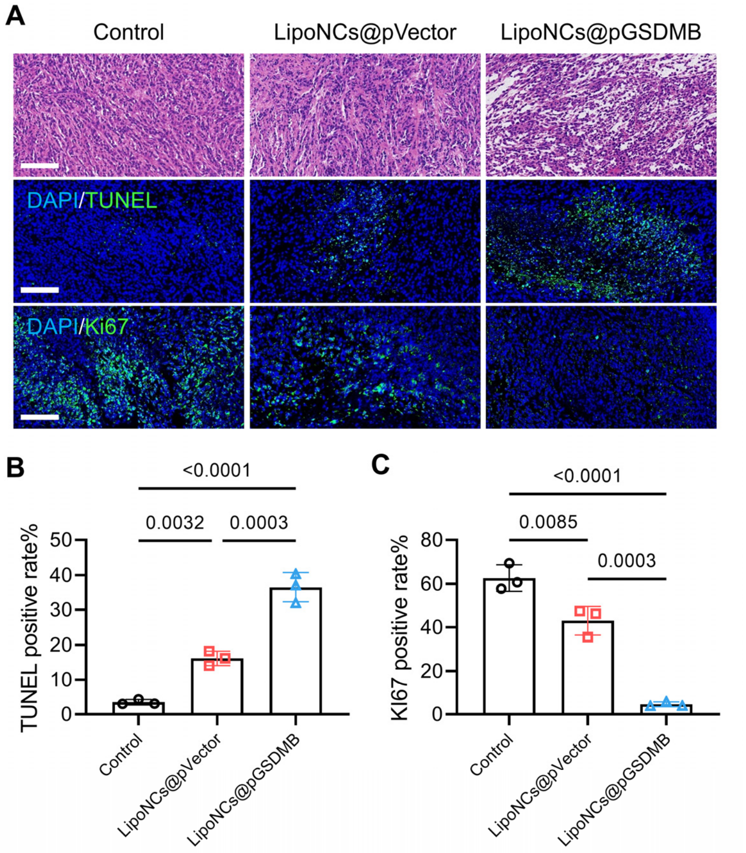

fig. S32. Tumor-suppressive and pro-apoptotic effects of LipoNCs@pGSDMB. (**A**) Representative images of tumor tissues from different treatment groups, showing hematoxylin and eosin (H&E) staining for histopathological assessment, TUNEL staining for apoptotic activity, and Ki67 immunostaining for proliferative activity. Scale bar, 100 μm. (**B**) Quantification of TUNEL-positive cells in tumor tissues. (**C**) Quantification of Ki67-positive cells in tumor tissues. Significant differences among groups were analyzed using one-way ANOVA. Data are presented as mean ± SD; n = 3.

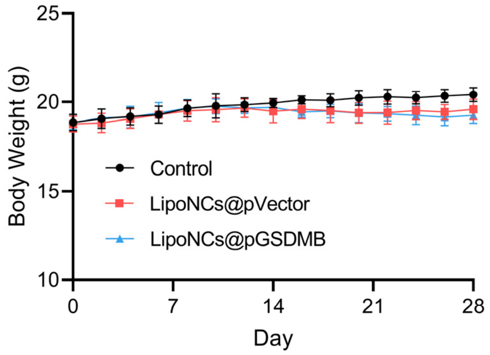

fig. S33. Body weight changes in mice during the experiment period (n = 7).

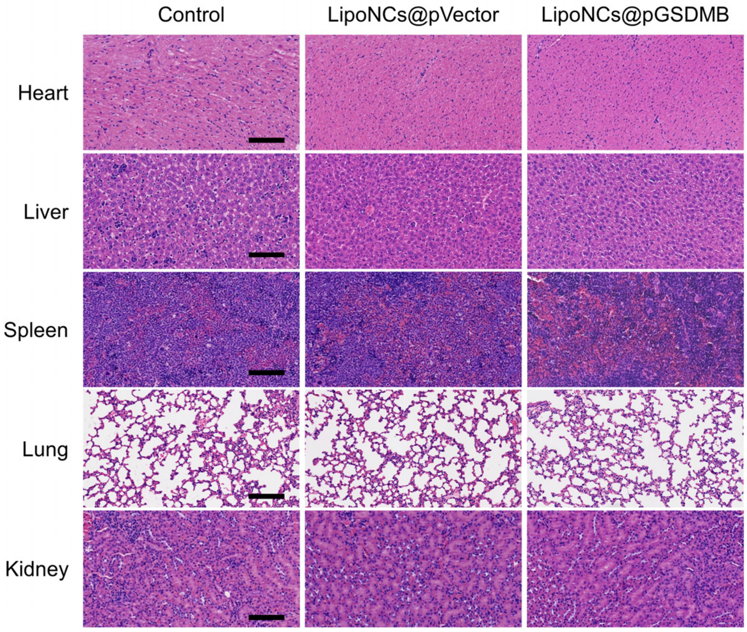

fig. S34. Assessment of major organ toxicity. Hematoxylin and eosin (H&E) staining was performed on major organs, including the heart, liver, spleen, lung, and kidney, obtained from treated mice. Scale bar, 100 μm.

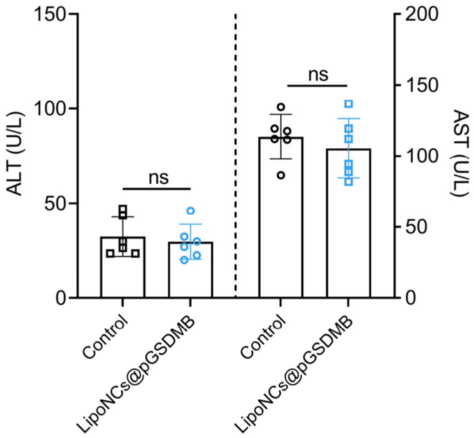

fig. S35. Assessment of hepatic toxicity of LipoNCs@pGSDMB. Serum alanine aminotransferase (ALT) and aspartate aminotransferase (AST) levels were measured in mice from the control and LipoNCs@pGSDMB groups at the 4-week endpoint; n = 6.

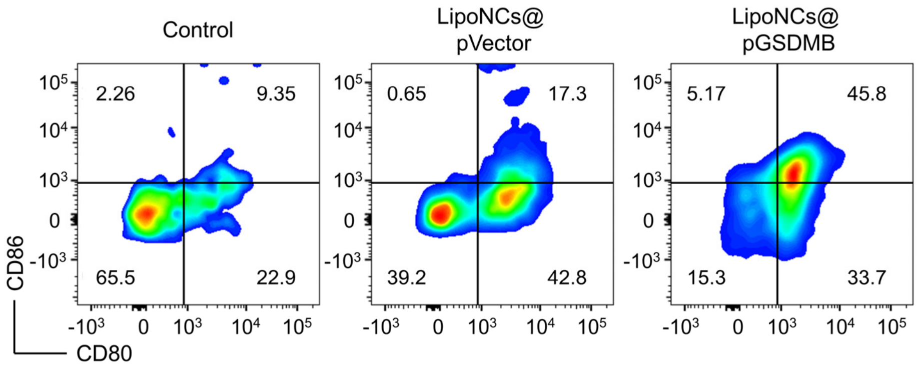

fig. S36. Flow cytometry analysis of mature dendritic cells (mDCs, CD11$^+$CD80$^+$CD86$^+$) in the tumor-draining lymph nodes.

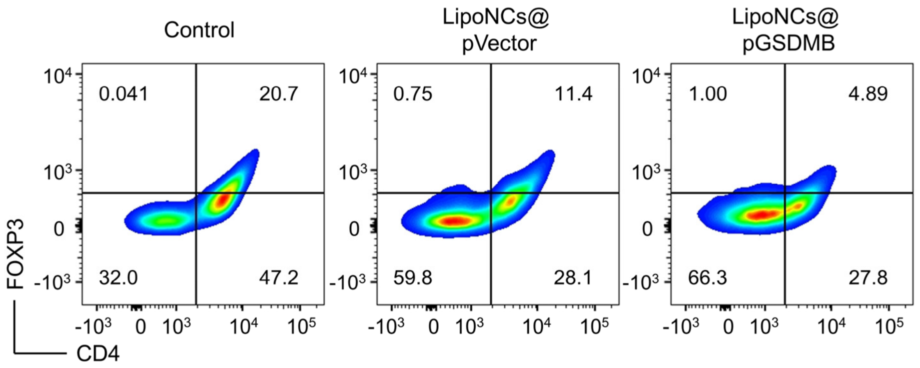

fig. S37. Flow cytometry analysis of regulatory T cells (Tregs, FOXP3$^+$CD4$^+$) in the tumor tissues.

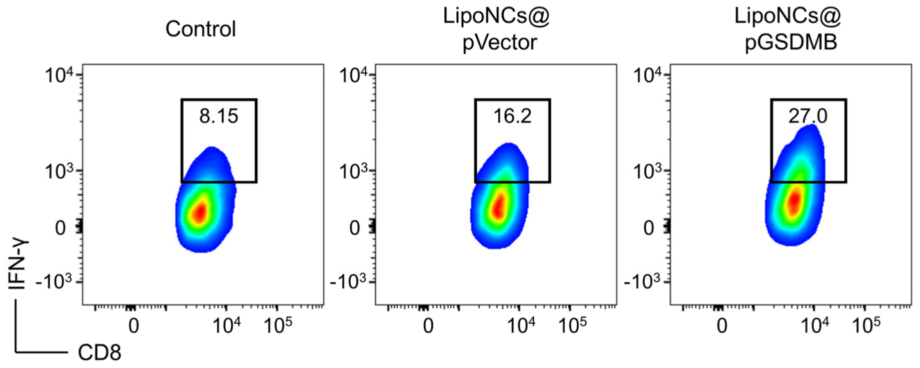

fig. S38. Flow cytometry analysis of activated CTLs (IFN-γ$^+$CD8$^+$) in the tumor tissues.

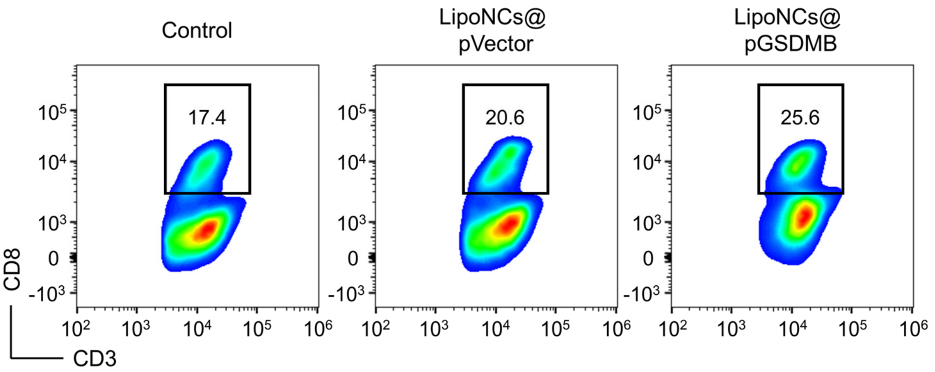

fig. S39. Flow cytometry analysis of CTLs (CD3⁺CD8⁺) in the spleens.

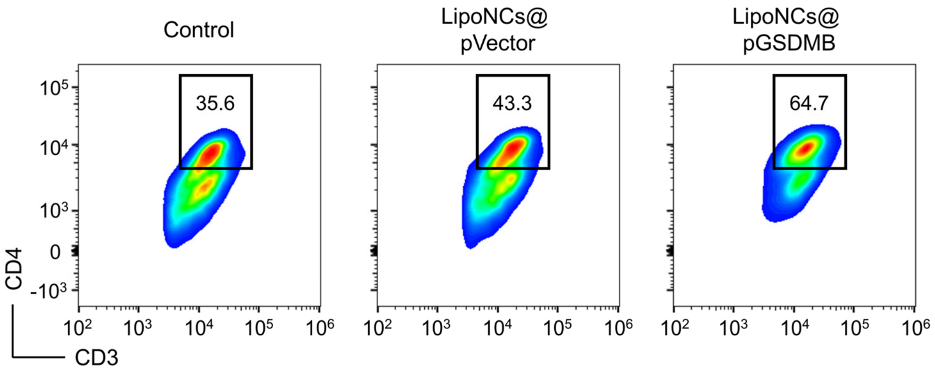

fig. S40. Flow cytometry analysis of Helper T cells (Ths, CD3⁺CD4⁺) in the spleens.

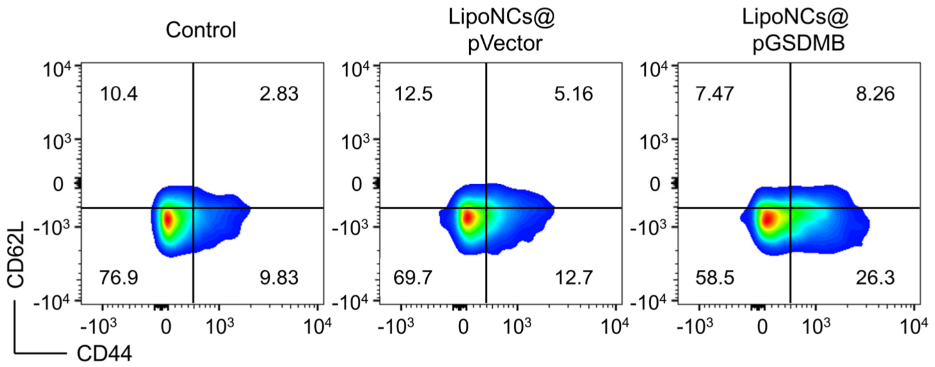

fig. S41. Flow cytometry analysis of central memory T cells (Tcms, CD44⁺CD62⁺) and effector memory T cells (Tems, CD44⁺CD62⁻) in the spleens.

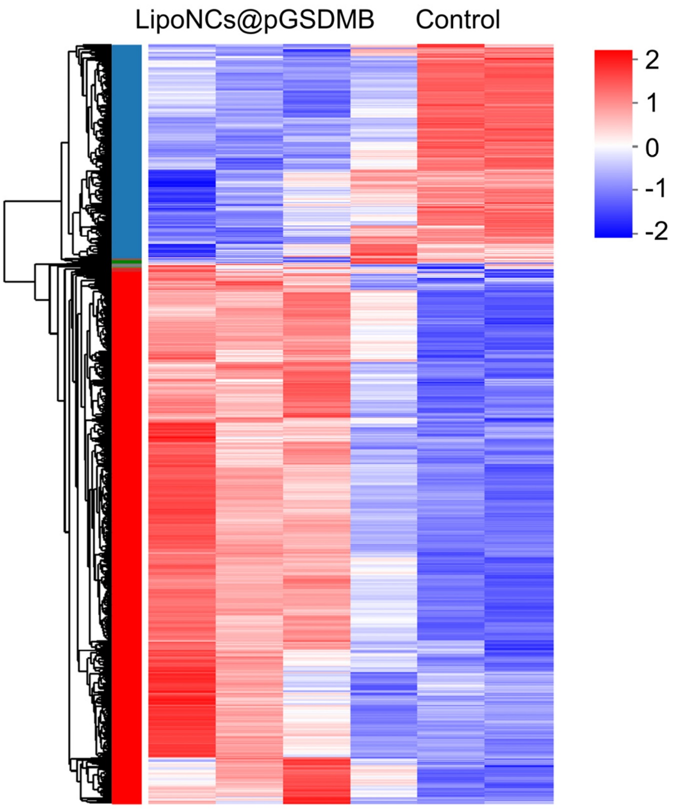

fig. S42. Differential gene expression in tumor cells after treatment. 4T1-GFP intra-femoral models were established and treated with PBS or LipoNCs@pGSDMB according to the regimen in Fig. 4A. GFP$^+$ tumor cells were isolated by flow cytometric sorting for bulk-seq at the 4-week endpoint. Genes with an adjusted P-value (<0.05) and an absolute log2 fold change (≥1) were considered differentially expressed. Gene expression differences are shown as $\log_2$ fold change ($\log_2$FC).

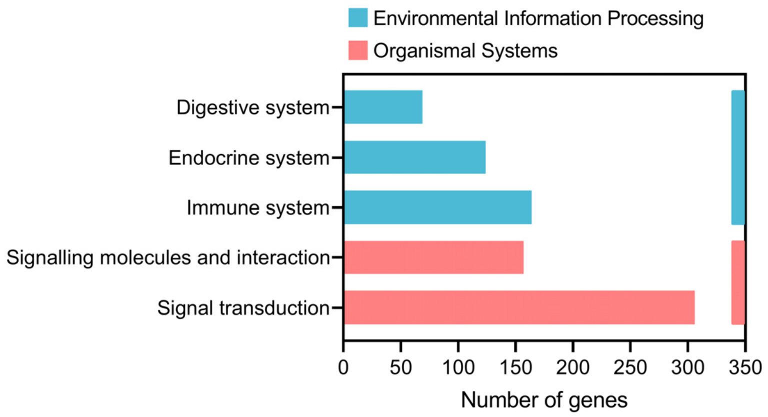

fig. S43. KEGG pathway classification for differentially expressed genes in tumor cells treated with LipoNCs@pGSDMB compared to the control group.

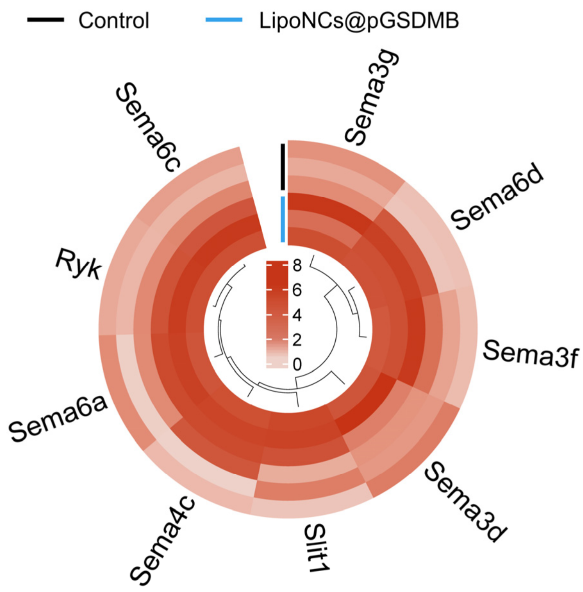

fig. S44. Circular heatmap illustrating the genes involved in the inhibition of axon guidance and tumor proliferation based on GO analysis. The inner and outer rings correspond to samples from the LipoNCs@pGSDMB and control groups, respectively.

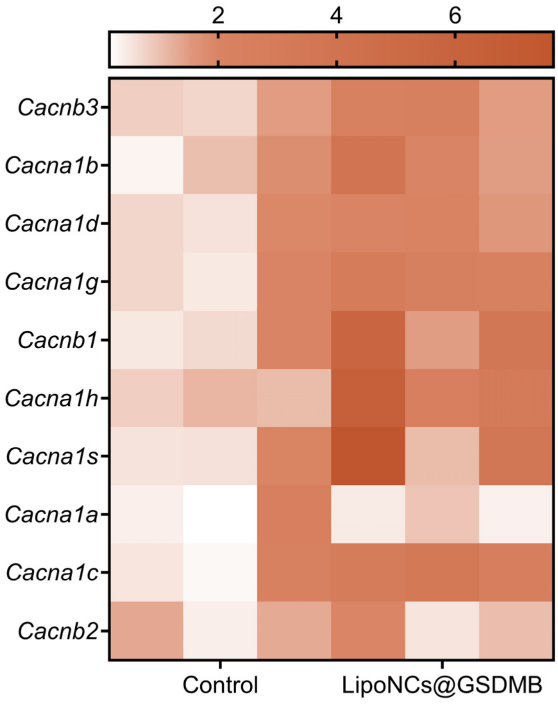

fig. S45. Heatmap illustrating the changes in VGCC-associated genes from the LipoNCs@pGSDMB and control groups.

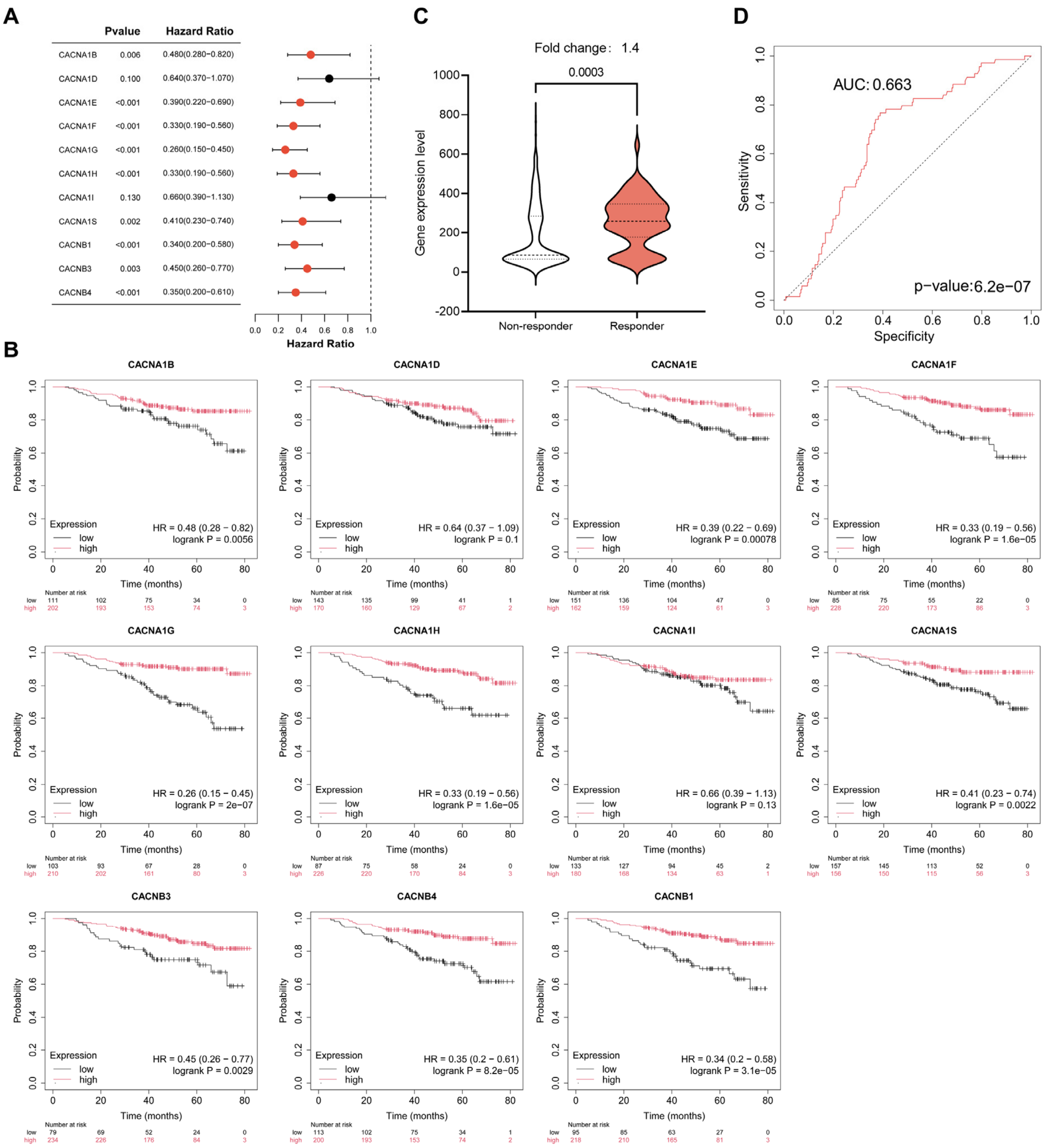

fig. S46. VGCC-related survival analysis and chemotherapy response assessment. (A) Forest plot showing hazard ratios and P values of VGCC-related genes in survival analysis. **(B)** Kaplan–Meier survival curves based on VGCC gene expression levels. **(C)** Differential expression of VGCCs between chemotherapy responders and non-responders. **(D)** ROC curve evaluating the predictive performance of VGCC expression for chemotherapy sensitivity.

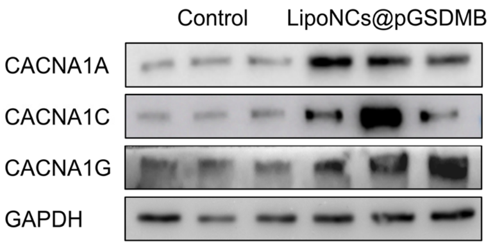

fig. S47. Assessment of VGCC expression after treatment. The expression levels of proteins related to VGCCs in tumor cells isolated from control or LipoNCs@pGSDMB-treated mice were evaluated using Western blot analysis, with GAPDH serving as the loading control.

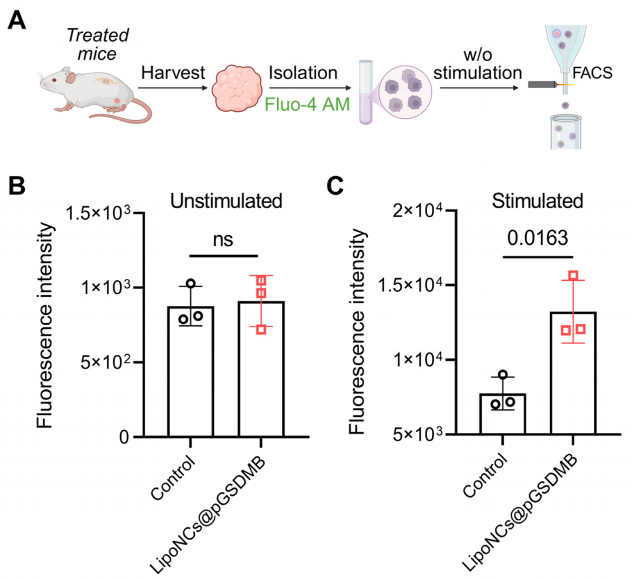

fig. S48. Flow cytometry analysis of calcium flux in primary tumor cells. (**A**) Schematic of calcium flux assay. "Created in BioRender. Zhang, Z. (2026) https://BioRender.com/23dd2vk "(**B, C**) Quantitative fluorescence intensity of calcium ion probes in tumor cells under resting (**B**) and KCl-stimulated conditions (**C**). Significant differences were assessed using Student's t-test. Data are presented as mean ± SD; n = 3.

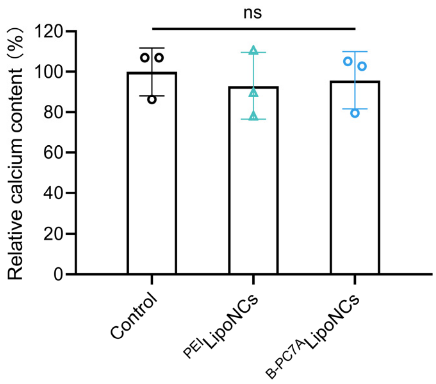

fig. S49. Quantitative analysis of intracellular calcium levels in 4T1 cells after treatment with ^{PEI}LipoNCs or ^{OPSA}LipoNCs for 48 hours. Calcium levels were measured using a Fluo-4 AM probe, and significant differences were assessed using one-way ANOVA. Data are presented as the mean ± SD; n = 3.

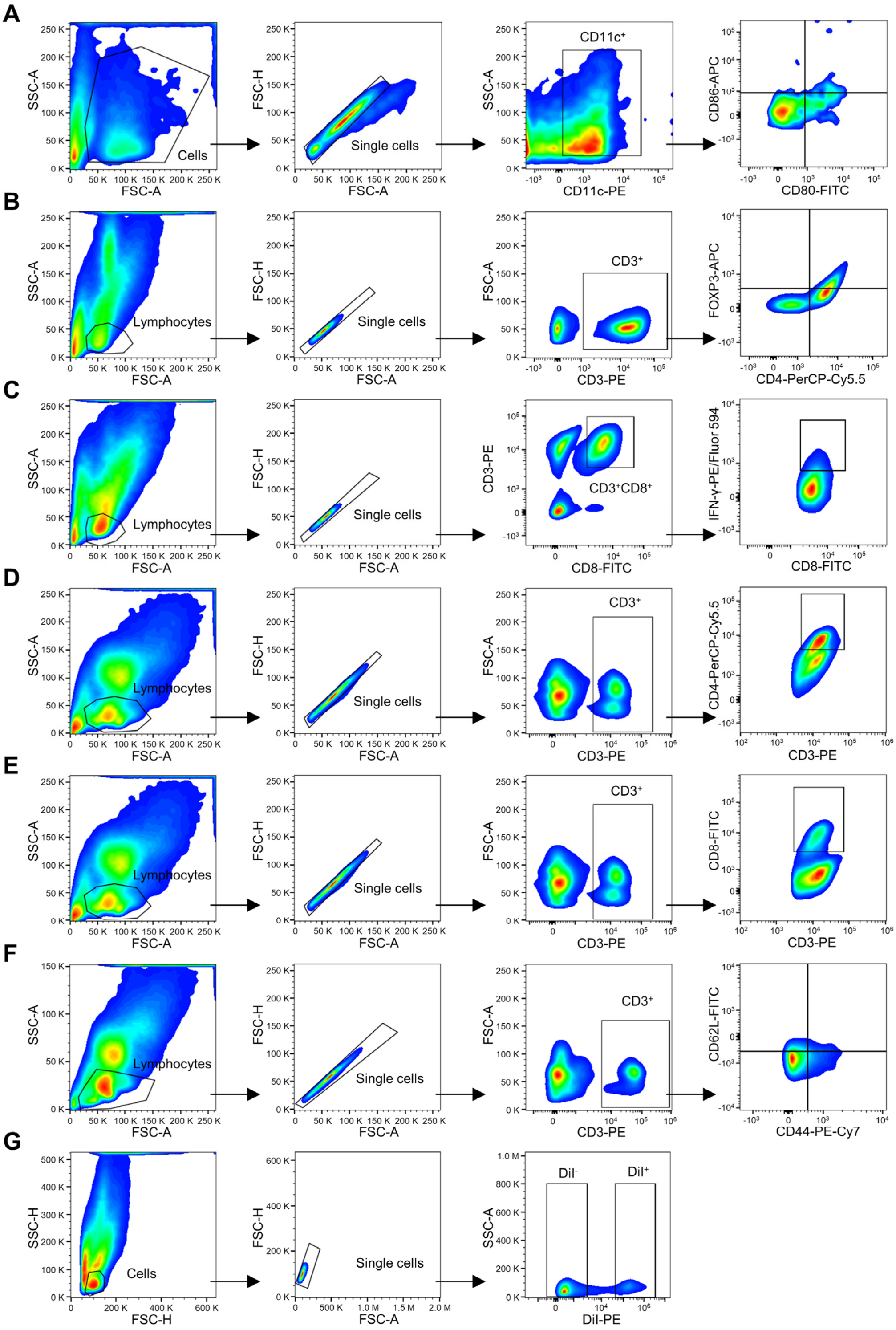

fig. S50. Flow cytometry gating strategies. Gating strategies to identify mDCs ($CD11^{+}CD80^{+}CD86^{+}$) (**A**), Tregs ($FOXP3^{+}CD4^{+}$) (**B**), activated CTLs ($IFN\text{-}\gamma^{+}CD8^{+}$) (**C**), Ths ($CD3^{+}CD4^{+}$) (**D**), CTLs ($CD4^{+}CD8^{+}$) (**E**), Tcms ($CD44^{+}CD62^{+}$), Tems ($CD44^{+}CD62^{-}$) (**F**), and DRG neurons (DiI^{+}) and tumor cells (DiI^{-}) (**G**).

table S1. Primer sequences

Primers	Sequences
CACNB2 F	CATCACCCCACTCCAAAGAGA
CACNB2 R	CGCCCTTCAAATCTGTGTTTTAG
CACNB4 F	TACCTGCATGGAGTTGAAGACT
CACNB4 R	TTCGCTCTCTCAAGCTGGATA
CACNA1A F	AGGCGATGATCCCTCTGCT
CACNA1A R	CCCTTCCCAGTACGGCTGA
CACNA1B F	TCTGAGCGACTAGATGACACG
CACNA1B R	GTTCCGAAGGTAGGAGCCC
CACNA1C F	TCCCGAGCACATCCCTACTC
CACNA1C R	ACTGACGGTAGAGATGGTTGC
CACNA1E F	CAGTCCGGCAGAACTGTTTCA
CACNA1E R	GAGACATTGGGGTCTTGTCATC
CACNA1F F	TACTAATCCCATTCGTCGGTCC
CACNA1F R	CATAGGCTACGATCTTGAGCAC
CACNA1G F	TGTCTCCGCACGGTCTGTAA
CACNA1G R	AAGCCGGTTCCAAGTGTCTC
CACNA1H F	ATGCTTGGGAACGTGCTTCTT
CACNA1H R	GTCTGGTAGTATGGCCGCAA
CACNA1I F	GGGCGTGGCCTGTTTAGTC
CACNA1I R	TGAGGGTCTCGGAGTGCTC
CACNA1S F	TCAGCATCGTGGAATGGAAAC
CACNA1S R	GTTCAGAGTGTTGTTGTCATCCT
*Ifnb1*F	ATGAGTGGTGGTTGCAGGC
Ifnb1 R	TGACCTTTCAAATGCAGTAGATTCA
CXCL10 F	GGAGTGAAGCCACGCACAC
CXCL10 R	ATGGAGAGAGGCTCTCTGCTGT
CTSK F	GAGGAAATGCTGGACACCCA
CTSK R	TTCAGGGCTTTCTCGTTCCC
cFos F	TTTCAACGCCGACTACGAGG
cFos R	GTAAGTAGTGCAGCCCGGA
NFATc1 F	CAAGTTTCCACTCGGCCCTC
NFATc1 R	GAGTGCTATCGGTGGTCAGG
MMP9 F	CTGGACAGCCAGACACTAAAG
MMP9 R	CTCGCGGCAAGTCTTCAGAG
ATP6v0d2 F	CAGAGCTGTACTTCAATGTGGAC
ATP6v0d2 R	AGGTCTCACACTGCACTAGGT
IL18 F	AGCAGTCCCAACTAAGCAGTA

IL18 R	CAGCCAGTAGAGGATGCTGA
IL1β F	GAAATGCCACCTTTTGACAGTG
IL1β R	TGGATGCTCTCATCAGGACAG
PTHrP F	CATCAGCTACTGCATGACAAGG
PTHrP F	GGTGGTTTTTGGTGTTGGGAG
RANKL F	CAGCATCGCTCTGTTCCTGTA
RANKL R	CTGCGTTTTCATGGAGTCTCA

REFERENCES

1. K. N. Weilbaecher, T. A. Guise, L. K. McCauley, Cancer to bone: A fatal attraction. *Nat. Rev. Cancer* **11**, 411–425 (2011).

2. E. Svensson, C. F. Christiansen, S. P. Ulrichsen, M. R. Rørth, H. T. Sørensen, Survival after bone metastasis by primary cancer type: A Danish population-based cohort study. *BMJ Open* **7**, e016022 (2017).

3. J. Zhang, D. Cai, S. Hong, Prevalence and prognosis of bone metastases in common solid cancers at initial diagnosis: A population-based study. *BMJ Open* **13**, e069908 (2023).

4. L. C. Hofbauer, A. Bozec, M. Rauner, F. Jakob, S. Perner, K. Pantel, Novel approaches to target the microenvironment of bone metastasis. *Nat. Rev. Clin. Oncol.* **18**, 488–505 (2021).

5. R. L. Satcher, X. H. Zhang, Evolving cancer-niche interactions and therapeutic targets during bone metastasis. *Nat. Rev. Cancer* **22**, 85–101 (2022).

6. B. March, S. Faulkner, P. Jobling, A. Steigler, A. Blatt, J. Denham, H. Hondermarck, Tumour innervation and neurosignalling in prostate cancer. *Nat. Rev. Urol.* **17**, 119–130 (2020).

7. P. Mantyh, Bone cancer pain: Causes, consequences, and therapeutic opportunities. *Pain* **154**, S54–S62 (2013).

8. D. D. Shi, J. A. Guo, H. I. Hoffman, J. Su, M. Mino-Kenudson, J. L. Barth, J. M. Schenkel, J. S. Loeffler, H. A. Shih, T. S. Hong, J. Y. Wo, A. J. Aguirre, T. Jacks, L. Zheng, P. Y. Wen, T. C. Wang, W. L. Hwang, Therapeutic avenues for cancer neuroscience: Translational frontiers and clinical opportunities. *Lancet Oncol.* **23**, 62–74 (2022).

9. R. S. Banh, D. E. Biancur, K. Yamamoto, A. S. W. Sohn, B. Walters, M. Kuljanin, A. Gikandi, H. Wang, J. D. Mancias, R. J. Schneider, M. E. Pacold, A. C. Kimmelman, Neurons release serine to support mRNA translation in pancreatic cancer. *Cell* **183**, 1202–1218.e25 (2020).

10. H. Fan, X. Liang, Y. Tang, Neuroscience in peripheral cancers: Tumors hijacking nerves and neuroimmune crosstalk. *MedComm* **5**, e784 (2024).

11. Y. Zhang, C. Lin, Z. Liu, Y. Sun, M. Chen, Y. Guo, W. Liu, C. Zhang, W. Chen, J. Sun, R. Xia, Y. Hu, X. Yang, J. Li, Z. Zhang, W. Cao, S. Sun, X. Wang, T. Ji, Cancer cells co-opt nociceptive nerves to thrive in nutrient-poor environments and upon nutrient-starvation therapies. *Cell Metab.* **34**, 1999–2017.e10 (2022).

12. N. Kuol, L. Stojanovska, V. Apostolopoulos, K. Nurgali, Role of the nervous system in cancer metastasis. *J. Exp. Clin. Cancer Res.* **37**, 5 (2018).

13. K. D. Candido, T. M. Kusper, N. N. Knezevic, New cancer pain treatment options. *Curr. Pain Headache Rep.* **21**, 12 (2017).

14. C. Abdel Shaheed, C. Hayes, C. G. Maher, J. C. Ballantyne, M. Underwood, A. J. McLachlan, J. H. Martin, S. W. Narayan, M. A. Sidhom, Opioid analgesics for nociceptive cancer pain: A comprehensive review. *CA Cancer J. Clin.* **74**, 286–313 (2024).

15. D. A. Silverman, V. K. Martinez, P. M. Dougherty, J. N. Myers, G. A. Calin, M. Amit, Cancer-associated neurogenesis and nerve-cancer cross-talk. *Cancer Res.* **81**, 1431–1440 (2021).

16. A. Salvalaggio, L. Pini, A. Bertoldo, M. Corbetta, Glioblastoma and brain connectivity: The need for a paradigm shift. *Lancet Neurol.* **23**, 740–748 (2024).

17. V. Padmanaban, I. Keller, E. S. Seltzer, B. N. Ostendorf, Z. Kerner, S. F. Tavazoie, Neuronal substance P drives metastasis through an extracellular RNA-TLR7 axis. *Nature* **633**, 207–215 (2024).

18. Q. Zeng, I. P. Michael, P. Zhang, S. Saghafinia, G. Knott, W. Jiao, B. D. McCabe, J. A. Galván, H. P. C. Robinson, I. Zlobec, G. Ciriello, D. Hanahan, Synaptic proximity enables NMDAR signalling to promote brain metastasis. *Nature* **573**, 526–531 (2019).

19. Y. Li, M. Huang, M. Wang, Y. Wang, P. Deng, C. Li, J. Huang, H. Chen, Z. Wei, Q. Ouyang, J. Zhao, Y. Lu, S. Su, Tumor cells impair immunological synapse formation via central nervous system-enriched metabolite. *Cancer Cell* **42**, 985–1002.e18 (2024).

20. G. Pascual, D. Domínguez, M. Elosúa-Bayes, F. Beckedorff, C. Laudanna, C. Bigas, D. Douillet, C. Greco, A. Symeonidi, I. Hernández, S. R. Gil, N. Prats, C. Bescós, R. Shiekhattar, M. Amit, H. Heyn, A. Shilatifard, S. A. Benitah, Dietary palmitic acid promotes a prometastatic memory via Schwann cells. *Nature* **599**, 485–490 (2021).

21. Z. Xin, L. Qin, Y. Tang, S. Guo, F. Li, Y. Fang, G. Li, Y. Yao, B. Zheng, B. Zhang, D. Wu, J. Xiao, C. Ni, Q. Wei, T. Zhang, Immune mediated support of metastasis: Implication for bone invasion. *Cancer Commun.* **44**, 967–991 (2024).

22. J. Massagué, K. Ganesh, Metastasis-initiating cells and ecosystems. *Cancer Discov.* **11**, 971–994 (2021).

23. S. Li, B. Mirlekar, B. M. Johnson, W. J. Brickey, J. A. Wrobel, N. Yang, D. Song, S. Entwistle, X. Tan, M. Deng, Y. Cui, W. Li, B. G. Vincent, M. Gale, Jr., Y. Pylayeva-Gupta, J. P. Ting, STING-induced regulatory B cells compromise NK function in cancer immunity. *Nature* **610**, 373–380 (2022).

24. J. Li, M. J. Hubisz, E. M. Earlie, M. A. Duran, C. Hong, A. A. Varela, E. Lettera, M. Deyell, B. Tavora, J. J. Havel, S. M. Phyu, A. D. Amin, K. Budre, E. Kamiya, J. A. Cavallo, C. Garris, S. Powell, J. S. Reis-Filho, H. Wen, S. Bettigole, A. J. Khan, B. Izar, E. E. Parkes, A. M. Laughney, S. F. Bakhoum, Non-cell-autonomous cancer progression from chromosomal instability. *Nature* **620**, 1080–1088 (2023).

25. H. Mohammadpour, C. R. MacDonald, G. Qiao, M. Chen, B. Dong, B. L. Hylander, P. L. McCarthy, S. I. Abrams, E. A. Repasky, β2 adrenergic receptor-mediated signaling regulates the immunosuppressive potential of myeloid-derived suppressor cells. *J. Clin. Invest.* **129**, 5537–5552 (2019).

26. M. Yang, L. Tao, Y. Jiang, J. Yang, Y. Huo, D. Liu, J. Li, X. Fu, R. He, C. Lin, W. Liu, J. Zhang, R. Hua, Q. Li, S. Jiang, L. Hu, G. Tian, X. Zhang, N. Niu, P. Lu, J. Shi, G. Xiao, L. Wang, J. Xue, Z. Zhang, Y. Sun, Perineural invasion reprograms the immune microenvironment through cholinergic signaling in pancreatic ductal adenocarcinoma. *Cancer Res.* **80**, 1991–2003 (2020).

27. D. K. Sarkar, C. Zhang, S. Murugan, M. Dokur, N. I. Boyadjieva, M. Ortigüela, K. R. Reuhl, S. Mojtehedzadeh, Transplantation of β-endorphin neurons into the hypothalamus promotes immune function and restricts the growth and metastasis of mammary carcinoma. *Cancer Res.* **71**, 6282–6291 (2011).

28. M. Balood, M. Ahmadi, T. Eichwald, A. Ahmadi, A. Majdoubi, K. Roversi, K. Roversi, C. T. Lucido, A. C. Restaino, S. Huang, L. Ji, K. C. Huang, E. Semerena, S. C. Thomas, A. E. Trevino, H. Merrison, A. Parrin, B. Doyle, D. W. Vermeer, W. C. Spanos, C. S. Williamson, C. R. Seehus, S. L. Foster, H. Dai, C. J. Shu, M. Rangachari, J. Thibodeau, S. V. Del Rincon, R. Drapkin, M. Rafei, N. Ghasemlou, P. D. Vermeer, C. J. Woolf, S. Talbot, Nociceptor neurons affect cancer immunosurveillance. *Nature* **611**, 405–412 (2022).

29. Y. Hou, B. Lin, T. Xu, J. Jiang, S. Luo, W. Chen, X. Chen, Y. Wang, G. Liao, J. Wang, J. Zhang, X. Li, X. Xiang, Y. Xie, J. Wang, S. Peng, W. Lv, Y. Liu, H. Xiao, The neurotransmitter calcitonin gene-related peptide shapes an immunosuppressive microenvironment in medullary thyroid cancer. *Nat. Commun.* **15**, 5555 (2024).

30. I. E. Demir, H. Friess, G. O. Ceyhan, Neural plasticity in pancreatitis and pancreatic cancer. *Nat. Rev. Gastroenterol. Hepatol.* **12**, 649–659 (2015).

31. X. OuYang, X. Xu, Q. Qin, C. Dai, H. Wang, S. Liu, L. Hu, X. Xiong, H. Liu, D. Zhou, Manganese-based nanoparticle vaccine for combating fatal bacterial pneumonia. *Adv. Mater.* **35**, e2304514 (2023).

32. H. Yin, C. Xie, Z. Zuo, D. Xie, Q. Wang, A CTL-inspired killing system using ultralow-dose chemical-drugs to induce a pyroptosis-mediated antitumor immune function. *Adv. Mater.* **36**, e2309839 (2024).

33. B. A. Simms, G. W. Zamponi, Neuronal voltage-gated calcium channels: Structure, function, and dysfunction. *Neuron* **82**, 24–45 (2014).

34. M. Luo, H. Wang, Z. Wang, H. Cai, Z. Lu, Y. Li, M. Du, G. Huang, C. Wang, X. Chen, M. R. Porembka, J. Lea, A. E. Frankel, Y. X. Fu, Z. J. Chen, J. Gao, A STING-activating nanovaccine for cancer immunotherapy. *Nat. Nanotechnol.* **12**, 648–654 (2017).

35. C. Glorieux, S. Liu, D. Trachootham, P. Huang, Targeting ROS in cancer: Rationale and strategies. *Nat. Rev. Drug Discov.* **23**, 583–606 (2024).

36. Z. Zhou, H. He, K. Wang, X. Shi, Y. Wang, Y. Su, Y. Wang, D. Li, W. Liu, Y. Zhang, L. Shen, W. Han, L. Shen, J. Ding, F. Shao, Granzyme A from cytotoxic lymphocytes cleaves GSDMB to trigger pyroptosis in target cells. *Science* **368**, eaaz7548 (2020).

37. X. Liu, J. Xiang, D. Zhu, L. Jiang, Z. Zhou, J. Tang, X. Liu, Y. Huang, Y. Shen, Fusogenic reactive oxygen species triggered charge-reversal vector for effective gene delivery. *Adv. Mater.* **28**, 1743–1752 (2016).

38. H. Wang, W. You, F. Gao, L. Zhang, A. Shen, F. Wang, G. Chen, X. Nie, L. Xia, W. Huang, W. Zhang, L. Wang, C. Hong, Z. Zhang, Y. You, Direct cytosolic delivery of DNA by creating fast closable holes in the cell membrane. *Chem. Eng. J.* **455**, 140962 (2023).

39. D. S. Spencer, A. B. Shodeinde, D. W. Beckman, B. C. Luu, H. R. Hodges, N. A. Peppas, Cytocompatibility, membrane disruption, and siRNA delivery using environmentally responsive cationic nanogels. *J. Control. Release* **332**, 608–619 (2021).

40. A. T. Ritter, G. Shtengel, C. S. Xu, A. Weigel, D. P. Hoffman, M. Freeman, N. Iyer, N. Alivodej, D. Ackerman, I. Voskoboinik, J. Trapani, H. F. Hess, I. Mellman, ESCRT-mediated membrane repair protects tumor-derived cells against T cell attack. *Science* **376**, 377–382 (2022).

41. S. R. Pillai, M. Damaghi, Y. Marunaka, E. P. Spugnini, S. Fais, R. J. Gillies, Causes, consequences, and therapy of tumors acidosis. *Cancer Metastasis Rev.* **38**, 205–222 (2019).

42. G. D. Roodman, Biology of osteoclast activation in cancer. *J. Clin. Oncol.* **19**, 3562–3571 (2001).

43. K. Wang, C. R. Donnelly, C. Jiang, Y. Liao, X. Luo, X. Tao, S. Bang, A. McGinnis, M. Lee, M. J. Hilton, R. R. Ji, STING suppresses bone cancer pain via immune and neuronal modulation. *Nat. Commun.* **12**, 4558 (2021).

44. N. M. Haynes, T. B. Chadwick, B. S. Parker, The complexity of immune evasion mechanisms throughout the metastatic cascade. *Nat. Immunol.* **25**, 1793–1808 (2024).

45. Y. Song, L. Teng, Y. Chen, C. M. Dong, Glycopolypeptide coordinated nanovaccine: Fabrication, characterization, and antitumor immune response. *Chem. Bio. Eng.* **1**, 633–646 (2024).

46. A. C. Stubbs, K. S. Martin, C. Coeshott, S. V. Skaates, D. R. Kuritzkes, D. Bellgrau, A. Franzusoff, R. C. Duke, C. C. Wilson, Whole recombinant yeast vaccine activates dendritic cells and elicits protective cell-mediated immunity. *Nat. Med.* **7**, 625–629 (2001).

47. D. Amsen, K. van Gisbergen, P. Hombrink, R. A. W. van Lier, Tissue-resident memory T cells at the center of immunity to solid tumors. *Nat. Immunol.* **19**, 538–546 (2018).

48. W. Yang, R. Yuste, In vivo imaging of neural activity. *Nat. Methods* **14**, 349–359 (2017).

49. Y. V. Gorbunova, N. C. Spitzer, Dynamic interactions of cyclic AMP transients and spontaneous Ca^{2+} spikes. *Nature* **418**, 93–96 (2002).

50. F. A. Russell, R. King, S. J. Smillie, X. Kodji, S. D. Brain, Calcitonin gene-related peptide: Physiology and pathophysiology. *Physiol. Rev.* **94**, 1099–1142 (2014).

51. M. Iftinca, M. Defaye, C. Altier, TRPV1-targeted drugs in development for human pain conditions. *Drugs* **81**, 7–27 (2021).

52. P. W. Mantyh, S. D. Rogers, P. Honore, B. J. Allen, J. R. Ghilardi, J. Li, R. S. Daughters, D. A. Lappi, R. G. Wiley, D. A. Simone, Inhibition of hyperalgesia by ablation of lamina I spinal neurons expressing the substance P receptor. *Science* **278**, 275–279 (1997).

53. F. Bradke, J. W. Fawcett, M. E. Spira, Assembly of a new growth cone after axotomy: The precursor to axon regeneration. *Nat. Rev. Neurosci.* **13**, 183–193 (2012).

54. G. R. Monteith, N. Prevarskaya, S. J. Roberts-Thomson, The calcium-cancer signalling nexus. *Nat. Rev. Cancer* **17**, 367–380 (2017).

55. M. Kühl, L. C. Sheldahl, M. Park, J. R. Miller, R. T. Moon, The Wnt/Ca^{2+} pathway: A new vertebrate Wnt signaling pathway takes shape. *Trends Genet.* **16**, 279–283 (2000).

56. Y. Luo, D. Raible, J. A. Raper, Collapsin: A protein in brain that induces the collapse and paralysis of neuronal growth cones. *Cell* **75**, 217–227 (1993).

57. A. Göhrig, K. M. Detjen, G. Hilfenhaus, J. L. Körner, M. Welzel, R. Arsenic, R. Schmuck, M. Bahra, J. Y. Wu, B. Wiedenmann, C. Fischer, Axon guidance factor SLIT2 inhibits neural invasion and metastasis in pancreatic cancer. *Cancer Res.* **74**, 1529–1540 (2014).

58. S. Sharma, S. Y. Wu, H. Jimenez, F. Xing, D. Zhu, Y. Liu, K. Wu, A. Tyagi, D. Zhao, H. W. Lo, L. Metheny-Barlow, P. Sun, J. D. Bourland, M. D. Chan, A. Thomas, A. Barbault, R. B. D'Agostino, C. T. Whitlow, V. Kirchner, C. Blackman, B. Pasche, K. Watabe, Ca^{2+} and CACNA1H mediate targeted suppression of breast cancer brain metastasis by AM RF EMF. *EBioMedicine* **44**, 194–208 (2019).

59. J. J. Pancrazio, M. P. Viglione, I. A. Tabbara, Y. I. Kim, Voltage-dependent ion channels in small-cell lung cancer cells. *Cancer Res.* **49**, 5901–5906 (1989).

60. D. S. Chandrashekar, B. Bashel, S. A. H. Balasubramanya, C. J. Creighton, I. Ponce-Rodriguez, B. Chakravarthi, S. Varambally, UALCAN: A portal for facilitating tumor subgroup gene expression and survival analyses. *Neoplasia* **19**, 649–658 (2017).

61. D. S. Chandrashekar, S. K. Karthikeyan, P. K. Korla, H. Patel, A. R. Shovon, M. Athar, G. J. Netto, Z. S. Qin, S. Kumar, U. Manne, C. J. Creighton, S. Varambally, UALCAN: An update to the integrated cancer data analysis platform. *Neoplasia* **25**, 18–27 (2022).

62. K. Ding, F. Chen, N. Priedigkeit, D. D. Brown, K. Weiss, R. Watters, K. M. Levine, T. Heim, W. Li, J. Hooda, P. C. Lucas, J. M. Atkinson, S. Oesterreich, A. V. Lee, Single cell heterogeneity and evolution of breast cancer bone metastasis and organoids reveals therapeutic targets for precision medicine. *Ann. Oncol.* **33**, 1085–1088 (2022).

63. B. Győrffy, Survival analysis across the entire transcriptome identifies biomarkers with the highest prognostic power in breast cancer. *Comput. Struct. Biotechnol. J.* **19**, 4101–4109 (2021).

64. J. T. Fekete, B. Győrffy, ROCplot.org: Validating predictive biomarkers of chemotherapy/hormonal therapy/anti-HER2 therapy using transcriptomic data of 3,104 breast cancer patients. *Int. J. Cancer* **145**, 3140–3151 (2019).

65. A. Gengatharan, S. Malvaut, A. Marymonchyk, M. Ghareghani, M. Snapyan, J. Fischer-Sternjak, J. Ninkovic, M. Götz, A. Saghatelyan, Adult neural stem cell activation in mice is regulated by the day/night cycle and intracellular calcium dynamics. *Cell* **184**, 709–722.e13 (2021).

66. T. A. Weissman, P. A. Riquelme, L. Ivic, A. C. Flint, A. R. Kriegstein, Calcium waves propagate through radial glial cells and modulate proliferation in the developing neocortex. *Neuron* **43**, 647–661 (2004).

67. V. Kaltezioti, I. P. Foskolou, M. D. Lavigne, E. Ninou, M. Tsampoula, M. Fousteri, M. Margarity, P. K. Politis, Prox1 inhibits neurite outgrowth during central nervous system development. *Cell. Mol. Life Sci.* **78**, 3443–3465 (2021).

68. L. Wang, J. Ge, H. Han, Y. Jia, Y. Qin, Crosstalk between the nervous system and tumor microenvironment: Functional aspects and potential therapeutic strategies. *Cancer Lett.* **594**, 216986 (2024).

69. K. M. Garland, T. L. Sheehy, J. T. Wilson, Chemical and biomolecular strategies for STING pathway activation in cancer immunotherapy. *Chem. Rev.* **122**, 5977–6039 (2022).

70. K. Yang, W. Han, X. Jiang, A. Piffko, J. Bugno, C. Han, S. Li, H. Liang, Z. Xu, W. Zheng, L. Wang, J. Wang, X. Huang, J. P. Y. Ting, Y. X. Fu, W. Lin, R. R. Weichselbaum, Zinc cyclic di-

AMP nanoparticles target and suppress tumours via endothelial STING activation and tumour-associated macrophage reinvigoration. *Nat. Nanotechnol.* **17**, 1322–1331 (2022).

71. C. R. Donnelly, C. Jiang, A. S. Andriessen, K. Wang, Z. Wang, H. Ding, J. Zhao, X. Luo, M. S. Lee, Y. L. Lei, W. Maixner, M. C. Ko, R. R. Ji, STING controls nociception via type I interferon signalling in sensory neurons. *Nature* **591**, 275–280 (2021).

72. A. H. Zahalka, P. S. Frenette, Nerves in cancer. *Nat. Rev. Cancer* **20**, 143–157 (2020).

73. T. Heinrich, C. A. Hübner, I. Kurth, Isolation and primary cell culture of mouse dorsal root ganglion neurons. *Bio Protoc.* **6**, e1785 (2016).

74. M. Li, D. Yang, H. Yan, Z. Tang, D. Jiang, J. Zhang, Z. Chi, W. Nie, W. Zhen, W. Yu, S. Chen, Z. Wang, Q. Yu, X. Zhang, F. Yang, S. Fan, X. Lin, D. Wang, Gasdermin D maintains bone mass by rewiring the endo-lysosomal pathway of osteoclastic bone resorption. *Dev. Cell* **57**, 2365–2380.e8 (2022).

75. C. Xie, X. Mao, J. Huang, Y. Ding, J. Wu, S. Dong, L. Kong, G. Gao, C. Li, L. Wei, KOBAS 2.0: A web server for annotation and identification of enriched pathways and diseases. *Nucleic Acids Res.* **39**, 316–322 (2011).

76. R. Foty, A simple hanging drop cell culture protocol for generation of 3D spheroids. *J. Vis. Exp.* **51**, e2720 (2011).
